# Irradiated Liver Cancer Cell Vaccine Transfected With GM‐CSF Induces Specific and Long‐Lasting Anti‐tumour Immunity Through the Synergistic Effect of Oxidised mtDNA and GM‐CSF

**DOI:** 10.1111/cpr.70198

**Published:** 2026-04-08

**Authors:** Zhiruo Song, Yujie Jiang, Yu Zhang, Danyi Ao, Chunjun Ye, Xiya Huang, Yingqiong Zhou, Hanle Yang, Ruolan Xia, Jiayuan Ai, Dandan Wan, Aiping Tong, Yuquan Wei, Xuemei He, Aqu Alu, Xiawei Wei

**Affiliations:** ^1^ Laboratory of Aging Research and Cancer Drug Target, State Key Laboratory of Biotherapy and Cancer Center, National Clinical Research Center for Geriatrics, West China Hospital Sichuan University Chengdu China

**Keywords:** cell‐based vaccine, c‐GAS/STING pathway, GM‐CSF, immunotherapy, liver cancer, mtDNA

## Abstract

Liver cancer remains a leading cause of cancer‐related mortality worldwide, with limited durable responses to conventional therapies. Cancer vaccines utilising the immune system offer a promising alternative. Here, we developed a prophylactic whole‐cell tumour vaccine by engineering Hepa 1‐6 cells to express murine granulocyte‐macrophage colony‐stimulating factor (mGM‐CSF), and investigated its anti‐tumour efficacy and underlying mechanisms. The Hepa 1‐6‐mGM‐CSF vaccine provided complete and durable protection against both primary and rechallenge tumour inoculations. Hepa 1‐6‐mGM‐CSF immunisation robustly activated dendritic cells (DCs) and T cells in both lymph nodes and spleen, characterised by enhanced DC maturation and migration, as well as the differentiation of T cells toward cytotoxic and memory phenotypes. Meanwhile, the Hepa 1‐6‐mGM‐CSF vaccine remodelled the tumour microenvironment (TME) toward an immunostimulatory state. Critically, irradiation‐induced oxidative stress in mitochondria promoted the release of oxidised mitochondrial DNA (ox‐mtDNA), which subsequently activated the cGAS‐STING signalling pathway. Ox‐mtDNA synergized with vaccine‐secreted GM‐CSF to promote DC activation, antigen presentation and migration. In summary, our study demonstrates that the Hepa 1‐6‐mGM‐CSF vaccine elicits robust anti‐tumour immunity through the coordinated release of ox‐mtDNA and GM‐CSF, with ox‐mtDNA synergistically enhancing immune activation via the cGAS‐STING signalling pathway. Collectively, these findings highlight the Hepa 1‐6‐mGM‐CSF vaccine as a promising strategy for liver cancer management.

## Graphical abstract

Song et al. developed an mGM‐CSF‐loaded, liver cancer cell‐based vaccine that potently activated dendritic cells and elicited tumour‐specific T cell responses. Mechanistically, radiation‐induced oxidative damage to mitochondrial DNA in cancer cells triggers the cGAS‐STING pathway in DCs, which synergizes with mGM‐CSF‐driven JAK2‐STAT3 signalling to enhance the vaccine's anti‐tumour immunity.

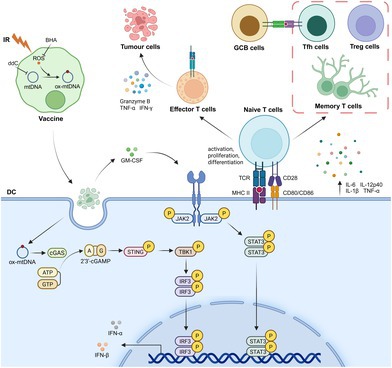

## 1 Introduction

Liver cancer, ranking the sixth most common cancer and the third leading cause of cancer‐related death worldwide, imposes a significant global health burden [1]. Primary liver cancer mainly comprises hepatocellular carcinoma (HCC) and intrahepatic cholangiocarcinoma (ICC), accounting for approximately 75%–85% and 10%–15% of liver cancer cases, respectively [1, 2]. For patients with early‐stage liver cancer, curative options include surgery, local ablation and liver transplantation [3, 4]. Patients with advanced liver cancer may receive intra‐arterial therapy, radiotherapy and systemic treatments such as tyrosine kinase inhibitors (sorafenib and lenvatinib) in the first‐line setting [4]. Despite these interventions, tumour recurrence remains a critical challenge. Even among patients with early‐stage, small HCC (< 3 cm), surgical resection is associated with unsatisfactory 5‐year survival rates of only 47%–53% [5]. Furthermore, patients with advanced‐stage HCC respond poorly to systemic therapies, resulting in an overall poor prognosis [6]. Therefore, there is an urgent need for novel and effective treatment approaches against liver cancer.

Immunotherapy has emerged as a transformative treatment strategy in oncology, eliminating malignant cells by activating the host immune system [7]. It encompasses multiple treatment strategies, such as immune checkpoint inhibitors (ICIs), adoptive cell therapy (ACT) and cancer vaccines, all of which have been extensively investigated in liver cancer. Among immunotherapies, tumour vaccines possess distinctive advantages in inducing specific anti‐tumour immunity through effective and comprehensive antigen presentation [8]. In HCC, ongoing research focuses on whole cell–based vaccines and peptide vaccines targeting antigens like morphogen receptor glypican‐3 (GPC3) [9]. Unlike vaccines based on individual specific antigens, whole tumour cell–based vaccines cover all potential antigens, thereby circumventing HLA restriction and reducing the risk of immune escape due to antigen loss [10]. To date, several whole tumour cell–based vaccines have been evaluated in clinical trials. Examples include vaccines combining inactivated autologous tumour cells with Bacillus Calmette‐Guérin (BCG) for the treatment of colon cancer or melanoma, as well as gemogenovatucel‐T, an engineered autologous tumour cell vaccine, developed for ovarian cancer [11, 12, 13]. However, investigations into cell‐based vaccines for liver cancer remain limited, and no such products have yet advanced to clinical evaluation.

To render tumour cells both immunogenic and safe, live tumour cells are commonly treated by various methods, such as ionising radiation (IR), a technique that has been extensively explored in preclinical and clinical studies [11, 14, 15, 16]. As a typical source of oxidative stress, IR can directly induce explosive accumulation of reactive oxygen species (ROS) within tumour cells, leading to oxidative damage and release of mitochondrial DNA (mtDNA) [17]. Notably, oxidised mtDNA (ox‐mtDNA) of irradiated tumour cells has been proven to be an important damage‐associated molecular pattern (DAMP), activating key immune signalling pathways including nuclear factor‐κB (NF‐κB) pathway, NLRP3 inflammasome activation pathway and cyclic GMP–AMP synthase (cGAS)‐stimulator of interferon genes (STING) pathway [18, 19, 20]. Among these, the cGAS‐STING pathway is pivotal for initiating anti‐tumour immunity. Previous studies demonstrate that diverse strategies, including Mn galvanic cells, cobalt fluoride and irradiated tumour cells, activate the cGAS‐STING pathway to elicit robust immune responses and suppress primary tumour growth [20, 21, 22]. Notably, the anti‐tumour efficacy of irradiated tumour cell vaccines was reported to be partially attributed to the activation of the cGAS‐STING pathway in dendritic cells (DCs), an important type of antigen‐presenting cells (APCs) crucial for initiating antigen‐specific immunity and immune tolerance [20, 23]. Besides, granulocyte‐macrophage colony‐stimulating factor (GM‐CSF) is frequently incorporated into whole‐cell vaccine platforms to enhance vaccine immunogenicity, as it is essential for regulating DC differentiation, activation and migration to lymphoid organs [8, 24].

In the present study, we developed a novel liver cancer cell‐based vaccine by genetically engineering Hepa 1‐6 cells to express murine GM‐CSF (Hepa 1‐6‐mGM‐CSF vaccine). We systematically evaluated its prophylactic efficacy and investigated the immunological mechanisms underlying vaccine‐induced anti‐tumour immunity in mouse models. Our findings demonstrate that immunisation with the Hepa 1‐6‐mGM‐CSF vaccine elicits robust immune responses, delays tumour progression, prolongs survival and provides long‐lasting protection against tumour recurrence. Mechanistically, the anti‐tumour efficacy of the vaccine is mediated by the synergistic activation of oxidised mtDNA‐induced STING signalling and the immunomodulatory effects of GM‐CSF secreted by the engineered tumour cells. Together, these results highlight the therapeutic potential of this vaccine platform and provide a strong rationale for its further development in clinical settings.

## 2 Materials and Methods

### 2.1 Mice and Cell Lines

Female C57BL/6 mice aged 6–8 weeks were purchased from Vital River (Peking, China). All animal experiments were approved by the Institutional Animal Care and Use Committee of Sichuan University (Chengdu, Sichuan, China). The mouse hepatoma cell line Hepa 1‐6 and the human kidney cell line HEK293T were purchased from the American Type Culture Collection (ATCC, Manassas, VA, USA). These cells were cultured in Dulbecco's modified Eagle's medium (DMEM; Gibco) or RPMI 1640 (Gibco) along with 10% fetal bovine serum (FBS; Corning), penicillin (100 U/mL) and streptomycin (100 mg/mL) at 37°C under 5% CO_2_ in a humidified atmosphere.

### 2.2 Vaccine Preparation

Recombinant lentivirus particles were produced in HEK293T cells by co‐transfection of three plasmids, including a plasmid containing the murine GM‐CSF (mGM‐CSF) gene and two packaging plasmids, pSPAX2 and pMD2.G. After filtration and concentration, the obtained lentivirus was used to infect Hepa 1‐6 cells. The infected Hepa 1‐6 cells were selected using puromycin, resulting in clones that stably express mGM‐SCF. These clones and Hepa 1‐6 cells transfected with an empty vector were irradiated with 100 Gy X‐ray and processed into vaccines for subsequent studies.

### 2.3 Reverse Transcription‐Quantitative PCR (RT‐qPCR) for Gene Expression

RNA was extracted from treated cells using the RNA Extraction Kit (TianGen, #DP419), and complementary DNA was generated using a PrimeSript RT reagent kit (Takara, #RR036) following the manufacturer's protocols. RT‐qPCR analysis was performed using SsoFast EvaGreen (Bio‐Rad, #1725202) on a Bio‐Rad iCycler RT–PCR detection system. The gene expression results were normalised to *β‐actin* (Actb) and determined by the 2^−ΔΔCt^ method (2^−ΔΔCt^). All primer sequences are listed in Table S1.

### 2.4 Immunisations and Tumour Models

For vaccination, 5 × 10^5^ irradiated Hepa 1‐6 cells or Hepa 1‐6‐mGM‐CSF cells were injected subcutaneously in the right flank of the dorsum on 0, 14 and 28 days. Mice in the control group were administered sterile phosphate‐buffered saline (PBS). Fourteen days after the final vaccination, mice were challenged with 5 × 10^6^ Hepa 1‐6 cells subcutaneously. For the tumour rechallenge model, 70 days after complete tumour regression, the immunised mice were subcutaneously re‐inoculated with 1.5 × 10^7^ Hepa 1‐6 cells. Tumour volume was measured every 3 days starting 7 days after tumour implantation and calculated using the following formula: volume (mm^3^) = length × width^2^ × 0.52.

### 2.5 Flow Cytometry (FCM)

Cells from the inguinal lymph nodes and spleens of immunised mice were harvested by grinding and filtering through a 70 μM strainer (Falcon). Red blood cells (RBC) were removed with RBC lysis buffer. The collected cells were then washed and resuspended in ice‐cold PBS. Tumour tissues were cut into small pieces and digested in RPMI 1640 medium (Gibco) with 1 mg/mL collagenase type I (Sigma‐Aldrich), 0.5 mg/mL collagenase Type IV (Sigma‐Aldrich) and 40 U/mL DNase I (Sigma), followed by incubation for 1 h at 37°C in a shaking incubator. The digested tumour tissues were subsequently filtered through 70 μm cell strainers, followed by red blood cell lysis, washing and resuspension in PBS to obtain single‐cell suspension.

For surface staining, cells were incubated with the indicated antibodies (Table S2, all from BioLegend, 1:100) at 4°C for 30 min. For intracellular staining, cells were pre‐treated with Brefeldin A (BD Biosciences) for 6 h to inhibit cytokine secretion. Then, the cells were collected and stained with surface markers as well as live/dead staining dye (Invitrogen, 1:1000). After being fixed and permeabilized with BD Cytofix/Cytoperm Fixation/Permeabilization Kit (BD Biosciences), the cells were incubated with intracellular antibodies (all from BioLegend, 1:100) overnight at 4°C. To evaluate the induced immune responses by vaccines, spleen lymphocytes were isolated and co‐cultured with irradiated Hepa 1‐6 cells (100 Gy) for 48 h. After incubation, cells were collected and stained with surface and intracellular antibodies based on the steps mentioned above. FCM was performed by Novocyte (Agilent), and data were analysed by NovoExpress software (1.5.6, ACEA Biosciences Inc., San Diego, CA). The key gating strategies are shown in Figures S1 and S2.

### 2.6 Spleen Lymphocytes Proliferation Assay

Spleen lymphocytes of immunised mice were isolated with density gradient centrifugation assay with Lymphocyte Separation Medium (Dakewe, China). The harvested lymphocytes were labelled with 2.5 μM Carboxyfluorescein succinimidyl ester (CFSE, Invitrogen, #C34554) at 37°C for 15 min in the dark. After incubation, lymphocytes were washed twice in PBS containing 5% FBS to remove excess CFSE. Splenic lymphocytes (1 × 10^6^ cells/well) were resuspended in RPMI 1640 supplemented with 10% FBS, plated in 12‐well plates and co‐cultured for 72 h with 1 × 10^5^ Hepa 1‐6 cells inactivated by 100 Gy X‐ray irradiation. The cells were then collected and the CFSE fluorescence value was measured by FCM.

### 2.7 BMDCs Generation, Uptake of Apoptotic Cells by BMDCs and Cross‐Presentation Assay

Bone marrow‐derived dendritic cells (BMDCs) were prepared as previously described [25]. In brief, bone marrow cells were isolated from the femurs and tibiae of C57BL/6 mice and filtered through a 70 μM strainer. After RBC lysis and washes, the cells were cultured in RPMI‐1640 medium containing 10% FBS, 50 μmol/L β‐mercaptoethanol (Sigma‐Aldrich), 1 mmol/L sodium pyruvate (Gibco, USA), 20 ng/mL murine GM‐CSF (Pepro Tech) and 10 ng/mL IL‐4 (Sigma‐Aldrich) for 7 days. For the apoptotic assay, irradiated Hepa 1‐6 and Hepa 1‐6‐mGM‐CSF cells were stained with CFSE according to the manufacturer's instructions. After culturing for 24 h in vitro, irradiated CFSE‐labelled cells were co‐cultured with BMDCs at a 3:1 ratio for 3 h. Non‐irradiated cells were used as control. Then, cells were harvested and stained with live/dead staining dye, PerCP‐Cy5.5‐conjugated anti‐CD45 and APC‐conjugated anti‐CD11c antibodies. The uptake of apoptotic cells was determined with FCM. For the cross‐presentation assay, BMDCs were co‐cultured with tumour cells with different treatments at a 1:3 ratio for 24 h. Subsequently, lymphocytes were prepared from the spleen of C57BL/6 mice and added to BMDCs at a 10:1 ratio for 48 h. Finally, the cells were stained with live/dead staining dye, PerCP‐Cy5.5‐conjugated anti‐CD3, APC‐conjugated anti‐CD4, FITC‐conjugated anti‐CD8, BV510‐conjugated anti‐CD44 and intracellular antibody BV421‐conjugated anti‐IFN‐γ for FCM assessment.

### 2.8 Detection of DCs in the Skin and Lymph Nodes

Irradiated Hepa 1‐6 cells or Hepa 1‐6‐mGM‐CSF cells were injected subcutaneously in the dorsum or hind footpad. After 24 h, skin tissues and popliteal lymph nodes were collected. Skin tissues were cut into small pieces and digested in the previously described buffer for 1 h at 37°C. The resulting cell suspensions and ground popliteal lymph nodes mixture were passed through 70 μM strainers and washed in PBS. To investigate the effect of blocking STING signalling on BMDCs, mice were immunised with irradiated Hepa 1‐6‐mGM‐CSF cells with or without intraperitoneally administered STING inhibitor C‐176 (MCE, 13.4 mg/kg) [26]. The single‐cell suspensions from skin and popliteal lymph nodes were stained with PE‐conjugated anti‐CD45, PE‐Cy7‐conjugated anti‐MHC‐II, BV510‐conjugated anti‐CD11c, APC‐conjugated anti‐CD80, FITC‐conjugated anti‐CD86, BV421‐conjugated anti‐CD40 and PerCP‐Cy5.5‐conjugated anti‐CCR7 for the FCM assay.

### 2.9 Enzyme‐Linked Immunosorbent Assay (ELISA)

To assess the maturation status of BMDCs, the supernatant was collected after co‐culturing BMDCs with different stimulants. Cytokines secreted by BMDCs were measured by ELISA according to the manufacturer's protocol (Thermo Fisher Scientific). Plates were read at 450 nm using a microplate reader (Biotek, USA).

### 2.10 Detection of Cell Death

For in vitro experiments, Hepa 1‐6 cells were treated with 100 Gy X‐ray irradiation and cultured for 24 h. As for in vivo experiments, irradiated Hepa 1‐6 cells were intraperitoneally injected into C57BL/6 mice. Mice were euthanized 24 h later, and cells were collected from the peritoneal lavage fluid. Cell death was evaluated with FITC Annexin V Apoptosis Detection Kit I (BD Pharmingen) by FCM. Furthermore, irradiated Hepa 1‐6 cells were fixed with 2% paraformaldehyde (PFA), permeabilized with 0.2% Triton X‐100, and stained with rabbit anti‐caspase‐3 (Cell Signalling Technology, #9664, 1:400) and FITC goat anti‐rabbit IgG (Abcam, 1:1000) antibody before evaluation with FCM.

### 2.11 Detection of Cellular ROS and 8‐Hydroxy‐2′‐Deoxyguanosine (8‐OHdG) Content

Hepa 1‐6 cells were irradiated by 100 Gy X‐ray with or without concurrent 100 μM butylated hydroxyanisole (BHA) treatment [27]. Intracellular ROS expression of Hepa 1‐6 cells was determined by the ROS Assay Kit (Beyotime). To test intracellular 8‐OHdG concentrations, irradiated and non‐irradiated Hepa 1‐6 cells were stained with mouse anti‐8‐OHdG antibody (Abcam, ab62623, 1:500) and FITC goat anti‐mouse IgG (Abcam, 1:1000) antibody and evaluated by FCM.

### 2.12 Immunofluorescence Assay

Cells were fixed by 2% paraformaldehyde solution for 10 min, permeabilized by 0.2% Triton X‐100 for 10 min and blocked with 10% goat serum for 30 min at room temperature. Hepa 1‐6 cells were incubated with primary antibodies, including mouse anti‐8‐OHdG antibody (Abcam, ab62623, 1:500) and rabbit anti‐TOMM20 antibody (Abcam, ab186734, 1:250) overnight at 4°C. Finally, cells were stained with secondary antibodies for 1 h and stained with DAPI (Life Technologies) for 10 min in darkness at room temperature. The cells were observed under IXplore Spin confocal microscopy (Olympus, Tokyo, Japan).

### 2.13 Mitochondrial DNA Isolation and Transfection

Mitochondrial DNA was isolated from tumour cells according to the manufacturer's protocol of the mtDNA Isolation Kit (Abcam, #ab65321). The resulting mtDNA was purified by phenol‐chloroform extraction and isopropanol precipitation. BMDCs were transfected with mtDNA using Lipofectamine 3000 (Invitrogen) following the manufacturer's protocol.

### 2.14 Western Blot Assay

Oxidised mtDNA (ox‐mtDNA) was extracted from irradiated Hepa 1‐6 cells and suspended in sterile H_2_O. BMDCs were stimulated with 5 μg/mL mtDNA, 5 μg/mL ox‐mtDNA, 200 ng/mL murine GM‐CSF and 1 μg/mL lipopolysaccharides (LPS, Sigma‐Aldrich). Total proteins were extracted by ice‐cold radioimmunoprecipitation assay buffer (RIPA; Beyotime) containing proteinase inhibitors (Millipore) and phosphatase inhibitors (KeyGen). The obtained proteins were separated by SDS‐PAGE and subsequently transferred onto polyvinylidene difluoride (PVDF) membranes (Millipore). After blocking with 5% non‐fat milk, the membranes were probed with specific primary antibodies (listed in Table S2) overnight at 4°C, with GAPDH protein serving as the loading control. Following primary antibody incubation, the membranes were incubated with horseradish peroxidase (HRP)‐conjugated goat anti‐rabbit (Abcam; 1:5000) and goat anti‐mouse (Abcam; 1:10,000) secondary antibodies for 1 h at room temperature. Protein bands were shown using ChemiDoc Touch (Bio‐Rad), and quantitative analysis was performed with ImageJ software.

### 2.15 Statistical Analysis

Data were analysed by GraphPad Prism 9.5.0 (GraphPad, San Diego, CA) and shown as mean ± SEM. Statistical significance was assessed using unpaired two‐tailed Student's *t*‐tests, one‐way ANOVA, two‐way ANOVA and log‐rank tests. *p* < 0.05 was considered significant.

## 3 Results

### 3.1 Construction of the Hepa 1‐6‐mGM‐CSF Vaccine and Evaluation of Its Inhibitory Effect on Tumour Growth and Recurrence

**FIGURE 1:**
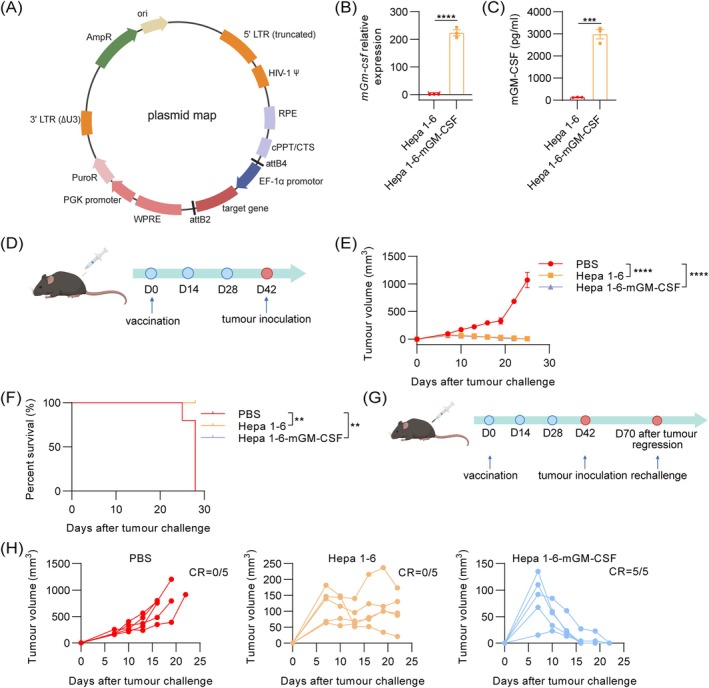
Vaccine construction and prophylactic animal experiment. (A) The schematic design of the mGM‐CSF plasmid. (B) Relative *mGm‐csf* mRNA levels in Hepa 1‐6 and Hepa 1‐6‐mGM‐CSF cells were measured by RT‐qPCR. (C) 5 × 10^5^ Hepa 1‐6 or Hepa 1‐6‐mGM‐CSF cells were seeded in six‐well plates. After culturing for 24 h, mGM‐CSF levels in the supernatants were evaluated with ELISA. (D) The timeline of the prophylactic animal experiment. (E) Kinetics of tumour growth and (F) overall survival in the prophylactic model. (G) The timeline of the tumour rechallenge experiment. (H) Kinetics of tumour growth in the tumour rechallenge model. CR, complete response. (B, C) *n* = 3 per group. (D–H) *n* = 5 per group. Data are shown as mean ± SEM and analysed with (B, C) unpaired two‐tailed Student's *t*‐tests, (E) two‐way ANOVA test and (F) log‐rank test. **p < 0.01, ***p < 0.001, ****p < 0.0001.

We constructed the recombinant plasmid of mGM‐CSF (Figure 1A) and generated lentivirus particles using the packaging plasmids, pSPAX2 and pMD2.G. The resulting lentivirus was then used to infect Hepa 1‐6 cells. Murine GM‐CSF expression in the infected cells was confirmed at both the RNA transcriptional and protein levels by RT‐qPCR and ELISA, respectively. Unmodified Hepa 1‐6 cells served as the negative control. As shown in Figure 1B, the engineered Hepa 1‐6‐mGM‐CSF cells successfully expressed the mGM‐CSF gene. Hepa 1‐6‐mGM‐CSF cells also secreted high levels of mGM‐CSF into the supernatant compared to unmodified Hepa 1‐6 cells (Figure 1C).

We next evaluated the anti‐tumour efficacy of different treatment regimens in the subcutaneous Hepa 1‐6 tumour model. On days 0, 14 and 28, mice were administered subcutaneously with PBS, 5 × 10^5^ irradiated Hepa 1‐6 cells, or 5 × 10^5^ irradiated Hepa 1‐6‐mGM‐CSF cells. On day 14 post the third immunisation, all mice were challenged subcutaneously with 5 × 10^6^ Hepa 1‐6 cells. By the end of the observation period, tumours in the Hepa 1‐6 and Hepa 1‐6‐mGM‐CSF groups had nearly disappeared, whereas all mice in the PBS group died by day 28 after tumour implantation (Figure 1E,F). On day 70 after complete tumour regression in mice following vaccination, the tumour‐eradicated mice were subcutaneously rechallenged with 1.5 × 10^7^ Hepa 1‐6 cells (Figure 1G). Notably, all the mice that received Hepa 1‐6‐mGM‐CSF vaccine completely rejected the re‐inoculated Hepa 1‐6 cells while Hepa 1‐6 vaccination did not (Figure 1H), demonstrating that the Hepa 1‐6‐mGM‐CSF vaccine provided robust and long‐lasting protection superior to conventional vaccine formulations. Moreover, we also evaluated the safety of Hepa 1‐6‐mGM‐CSF vaccine. On day 7 following the third immunisation, serum samples and major organs including heart, liver, spleen, lung and kidney were collected from the mice. Throughout the immunisation period, no adverse reactions such as hair loss, behavioural abnormalities, or significant changes in body weight were observed (Figure S3A). Furthermore, major organs showed no notable abnormalities, and serum biochemical parameters demonstrated no marked differences, indicating the vaccine had no adverse effects on the mice (Figure S3B,C). In summary, the Hepa 1‐6‐GM‐CSF vaccine displayed an excellent safety profile.

### 3.2 Hepa 1‐6‐mGM‐CSF Vaccine Immunisation Activated DC and T Cell Immunity in the Inguinal Lymph Nodes and Spleen

**FIGURE 2:**
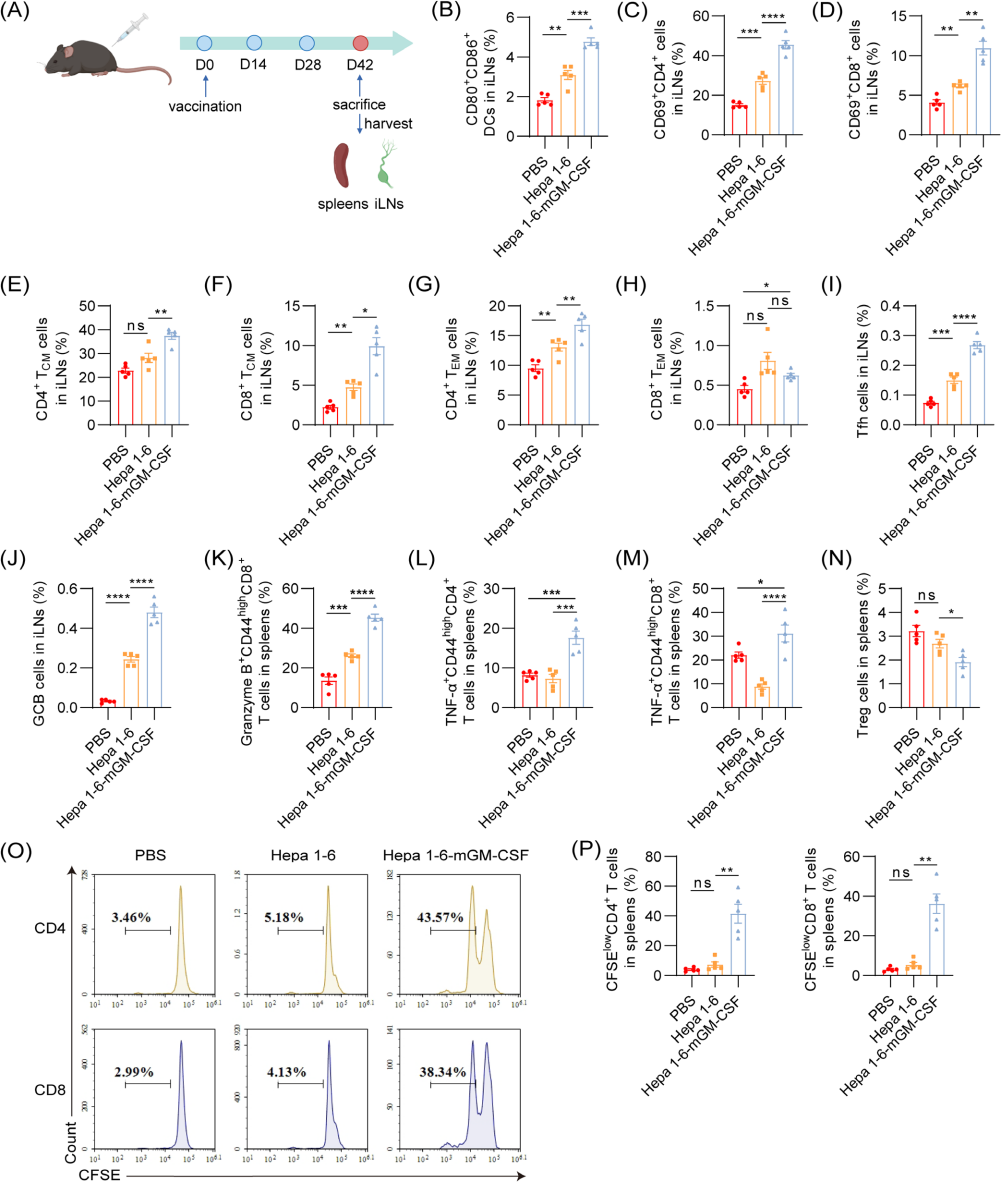
Hepa 1‐6‐mGM‐CSF vaccine activates DCs and T cell immunity in lymph nodes and spleens. (A) The timeline of prophylactic experiments. (B–J) Percentages of mature DCs (CD11c^+^CD80^+^CD86^+^, B), activated CD4^+^ (CD69^+^CD4^+^, C) and CD8^+^ (CD69^+^CD8^+^, D) T cells, CD4^+^ and CD8^+^ T_CM_ (CD44^+^CD62L^+^, E, F), CD4^+^ and CD8^+^ T_EM_ (CD44^+^CD62L^−^, G, H), Tfh cells (CXCR5^+^PD‐1^+^, I), GCB cells (CD95^+^GL‐7^+^, J) in the inguinal lymph nodes (iLNs). (K–M) Percentages of granzyme B‐producing CD44^high^CD8^+^ T cells (K), TNF‐α‐producing CD44^high^CD4^+^ (L) and CD44^high^CD8^+^ (M) T cells in splenic lymphocytes after stimulation with irradiated Hepa 1‐6 cells for 48 h. (N) Percentages of Treg cells (CD4^+^CD25^+^Foxp3^+^) in spleens. (O, P) Representative images (O) and quantifications (P) of the percentages of proliferative CD4^+^ and CD8^+^ T cells (CFSE^low^) in splenic lymphocytes after being stimulated by irradiated Hepa 1‐6 cells for 72 h. *n* = 5 per group. Data are shown as mean ± SEM and analysed with one‐way ANOVA test. *p < 0.05, **p < 0.01, ***p < 0.001, ****p < 0.0001, ns, not statistically significant.

We next evaluated the effect of the Hepa 1‐6‐mGM‐CSF vaccine on anti‐tumour immunity. Mice were injected subcutaneously with irradiated Hepa 1‐6‐mGM‐CSF cells, irradiated Hepa 1‐6 cells, or PBS on days 0, 14 and 28. On day 42, mice were euthanized and the inguinal lymph nodes (iLNs) and spleens were harvested to assess cellular immune responses. As the most efficient APCs, DCs attract naïve CD4^+^ and CD8^+^ T cells by secreting chemokines, and subsequently present antigens to and activate T cells through major histocompatibility complex (MHC) binding with T cell receptor (TCR) [28]. FCM analysis revealed a significant increase in the frequency of mature DCs in the iLNs of mice in the Hepa 1‐6‐mGM‐CSF group compared with mice immunised with PBS or irradiated Hepa 1‐6 cells, characterised by enhanced expression of the co‐stimulatory molecules CD80 and CD86 (Figure 2B). Meanwhile, Hepa 1‐6‐mGM‐CSF immunisation elicited the highest proportions of CD4^+^CD69^+^ and CD8^+^CD69^+^ T cells in the iLNs among different groups, indicating the activation of both CD4^+^ and CD8^+^ T cell immune responses (Figure 2C,D). Memory T cells, primarily categorised into central memory T (T_CM_) cells and effector memory T (T_EM_) cells, differentiate from naïve T cells [29]. Upon re‐exposure to the original stimulus, they can elicit sustained and enhanced immune responses [29]. Our results revealed a greater increase of CD4^+^ and CD8^+^ T_CM_ cells (CD44^+^CD62L^+^) and T_EM_ cells (CD44^+^CD62L^−^) in the Hepa 1‐6‐mGM‐CSF group than the other groups (Figure 2E–H), indicating the establishment of durable protective immunity. T follicular helper (Tfh) cells and germinal centre B (GCB) cells are crucial cells in humoral immunity. With the help of cytokines secreted by Tfh cells, GCB cells complete the class‐switch recombination (CSR) process and differentiate into plasma cells and memory B cells, thereby participating in humoral immunity [30]. In our study, we also found that the Hepa 1‐6‐mGM‐CSF vaccine stimulated the humoral arm of the adaptive immune system, as evidenced by expanded populations of Tfh (CXCR5^+^PD‐1^+^) and GCB (CD95^+^GL‐7^+^) cells (Figure 2I,J).

To further investigate T cell responses, splenic lymphocytes from the immunised mice were isolated and stimulated in vitro with irradiated Hepa 1‐6 cells for 48 h. Granzyme B and tumour necrosis factor‐α (TNF‐α) are important cytotoxic mediators secreted by T cells [28]. Upon activation via the TCR, naïve T cells exhibit increased CD44 expression, which is maintained at high levels in memory cells [31, 32]. Consequently, it is commonly used to identify T cells that have undergone antigen stimulation [31]. The Hepa 1‐6‐mGM‐CSF group exhibited the highest frequencies of granzyme B^+^ cells among CD44^high^CD8^+^ T cells and TNF‐α^+^ cells among CD44^high^CD4^+^ T cells or CD44^high^CD8^+^ T cells, suggesting that the Hepa 1‐6‐mGM‐CSF vaccine induced strong cytotoxic T cell immunity (Figure 2K–M). Regulatory T (Treg) cells can suppress excessive immune activation and maintain immune tolerance [33]. Our results showed that the Hepa 1‐6‐mGM‐CSF vaccine reduced the proportions of Treg cells (CD4^+^CD25^+^Foxp3^+^) in splenocytes, yet the Hepa 1‐6 vaccine couldn't (Figure 2N). Moreover, splenic CD4^+^ and CD8^+^ T cells from Hepa 1‐6‐mGM‐CSF‐vaccinated mice demonstrated stronger proliferative activity following 72 h of stimulation with irradiated Hepa 1‐6 cells (Figure 2O,P). Based on the results, we proposed that the Hepa 1‐6‐mGM‐CSF vaccine primed T cells in vivo, which induced recall immune responses upon re‐encountering the same tumour antigens. In summary, these diverse enhancing effects on cellular and humoral immunity, combined with the reduction of immunosuppressive cells, form the basis for outstanding anti‐tumour efficacy of the vaccine.

### 3.3 Hepa 1‐6‐mGM‐CSF Vaccine Reshapes the Tumour Microenvironment and Promotes Memory T Cell Immunity

**FIGURE 3:**
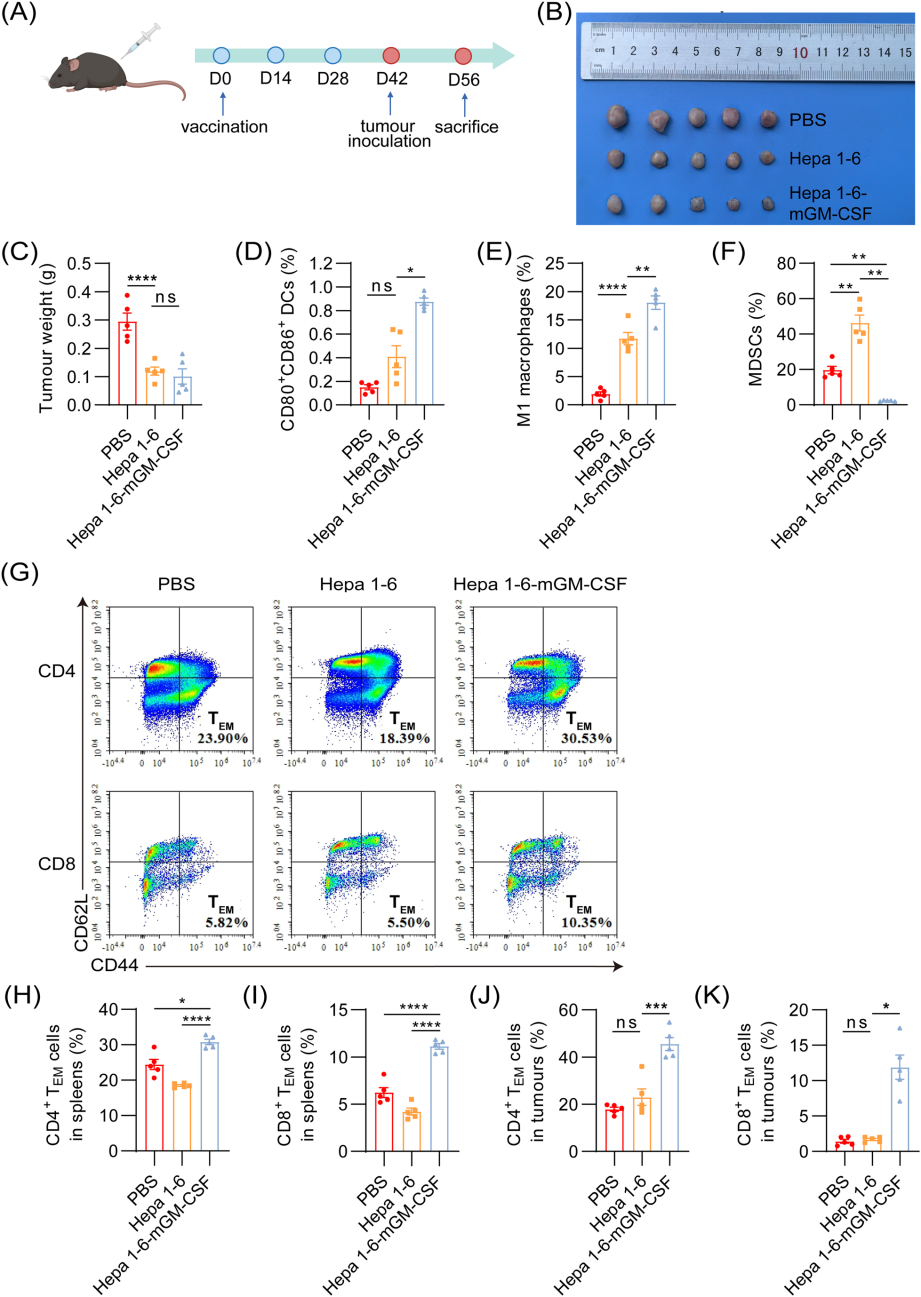
Hepa 1‐6‐mGM‐CSF vaccine reshapes TME and induces memory T cell immunity. (A) The timeline of experiments. Mice were treated with PBS, 5 × 10^5^ irradiated Hepa 1‐6 cells or Hepa 1‐6‐mGM‐CSF cells subcutaneously on days 0, 14 and 28. On day 42, mice were challenged with 5 × 10^6^ Hepa 1‐6 cells. On day 56, mice were euthanized, and tumour tissues and spleens were harvested. Representative images (B) and quantification of tumour weight (C) of resected tumours were displayed. (D–F) Percentages of mature DCs (D), M1 macrophages (F4/80^+^MHC‐II^+^, E) and MDSCs (CD11b^+^Gr‐1^+^, F) in tumour samples. (G–I) Representative FCM images (G) and quantifications of CD4^+^ (H) and CD8^+^ (I) T_EM_ frequencies in spleens. (J, K) Quantifications of CD4^+^ (J) and CD8^+^ (K) T_EM_ frequencies in the tumour samples. *n* = 5 per group. Data are shown as mean ± SEM and analysed with one‐way ANOVA test. *p < 0.05, **p < 0.01, ***p < 0.001, ****p < 0.0001, ns, not statistically significant.

To better understand the mechanisms of the anti‐tumour effect of the Hepa 1‐6‐mGM‐CSF vaccine, we immunised mice on days 0, 14 and 28 and challenged the vaccinated mice with Hepa 1‐6 tumour cells subcutaneously on day 42. The tumour and spleen samples from all groups were collected on day 56 to explore immune regulation in the tumour microenvironment (TME). Immunisation with both Hepa 1‐6‐mGM‐CSF vaccine and Hepa 1‐6 vaccine exhibited significant reductions in tumour growth and tumour weight compared to the PBS group (Figure 3B,C). TME refers to the microenvironment surrounding tumour cells, composed of cellular and non‐cellular components [34]. Tumour infiltrating cells include DCs, M1 macrophages with pro‐inflammatory and anti‐tumour functions, myeloid‐derived suppressor cells (MDSCs) associated with immune suppression, T cells etc. [34]. Critically, FCM analysis revealed that vaccination with Hepa 1‐6‐mGM‐CSF significantly promoted the infiltration of mature DCs into the TME compared with PBS immunisation, showing a higher tendency than the Hepa 1‐6 group (Figure 3D). Meanwhile, both Hepa 1‐6‐mGM‐CSF and Hepa 1‐6 vaccination promoted M1 macrophage (F4/80^+^MHC‐II^+^) polarisation in the TME, with a more pronounced increase observed in the Hepa 1‐6‐mGM‐CSF group (Figure 3E). Notably, the Hepa 1‐6 vaccine enhanced the local accumulation of immunosuppressive MDSCs (CD11b^+^Gr‐1^+^), while the Hepa 1‐6‐mGM‐CSF vaccine markedly decreased their proportion within the TME (Figure 3F).

We next determined the alterations in T cell immunity both in the TME and spleen of the tumour‐bearing mice. As shown in Figure 3G–I, pronounced elevations of CD4^+^ and CD8^+^ T_EM_ in the spleen were observed in mice immunised with the Hepa 1‐6‐mGM‐CSF vaccine. Consistently, Hepa 1‐6‐mGM‐CSF vaccination strongly elevated the frequencies of CD4^+^ and CD8^+^ T_EM_ in the tumour tissues (Figure 3J,K). In contrast, the Hepa 1‐6 vaccine exerted a limited effect on the induction of T_EM_ immunity in both the spleen and tumour tissues of the tumour‐bearing mice. This insufficient induction of memory T cell immunity may account for its limited efficacy in protecting against a secondary challenge with the same Hepa 1‐6 cells, as described above. In summary, the Hepa 1‐6‐mGM‐CSF vaccine induced robust antigen‐specific anti‐tumour immune responses, alleviated the immunosuppressive TME and conferred long‐term protective immunity. This shift in the immunophenotypic landscape indicates that the mGM‐CSF‐loaded vaccine effectively reprograms the TME toward an immunostimulatory state.

### 3.4 Hepa 1‐6‐mGM‐CSF Vaccine Enhances DC Maturation and Migration

**FIGURE 4:**
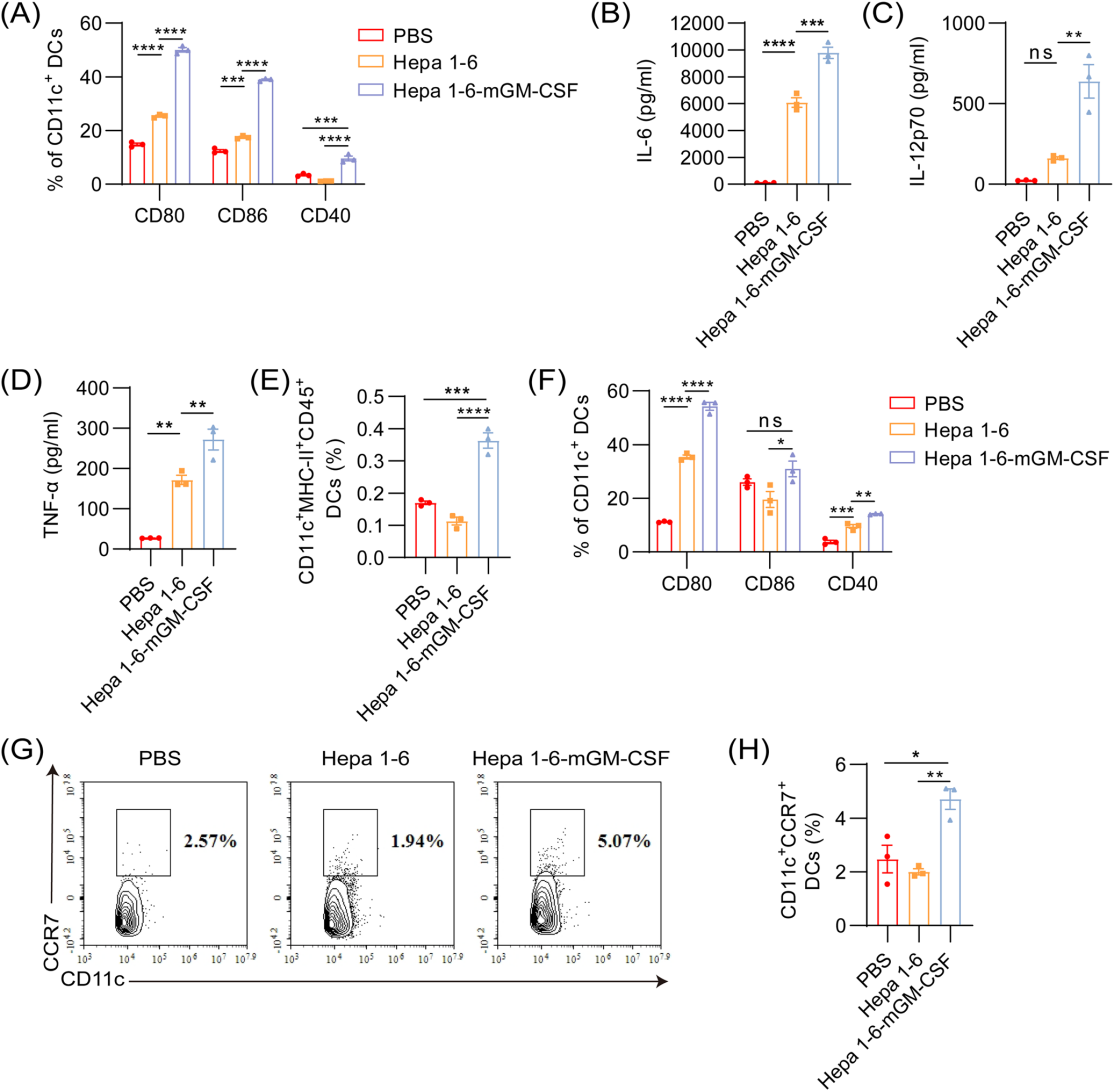
Hepa 1‐6‐mGM‐CSF vaccine promotes DC maturation and migration. (A–D) BMDCs were co‐cultured with PBS, irradiated Hepa 1‐6 or Hepa 1‐6‐mGM‐CSF cells for 24 h. (A) FCM was applied to evaluate the percentages of CD40, CD80 and CD86 positive cells among CD11c^+^ DCs. Supernatants were collected to measure levels of IL‐6 (B), IL‐12p70 (C) and TNF‐α (D) using ELISA. (E) Mice were injected with PBS, 5 × 10^5^ irradiated Hepa 1‐6 or Hepa 1‐6‐mGM‐CSF cells subcutaneously. After 24 h, the percentage of CD45^+^CD11c^+^MHC‐II^+^ cells within the skin was detected. (F–H) Mice were administered with PBS, 5 × 10^5^ irradiated Hepa 1‐6 or Hepa 1‐6‐mGM‐CSF cells in the hind footpad. Popliteal LNs were excised to evaluate the expression of CD40, CD80 and CD86 among CD11c^+^ DCs (F) and the percentage of CD11c^+^ CCR7^+^ cells (G, H). *n* = 3 per group. Data are shown as mean ± SEM and analysed with one‐way ANOVA test. *p < 0.05, **p < 0.01, ***p < 0.001, ****p < 0.0001, ns, not statistically significant.

Given the indispensable role of mGM‐CSF in DCs, we conducted a series of experiments to investigate the effects of the Hepa 1‐6‐mGM‐CSF vaccine on DC biology. For in vitro assays, BMDCs were co‐cultured with PBS, irradiated Hepa 1‐6 or Hepa 1‐6‐mGM‐CSF cells for 24 h. Our findings revealed that the Hepa 1‐6‐mGM‐CSF vaccine up‐regulated the expression of maturation markers (CD80, CD86 and CD40) on DCs and elevated the secretion of pro‐inflammatory cytokines, including IL‐6, IL‐12p70, and TNF‐α, which were significantly higher than those stimulated by the Hepa 1‐6 vaccine (Figure 4A–D). We next carried out an in vivo immunisation experiment to further verify the effect of the Hepa 1‐6‐mGM‐CSF vaccine on the recruitment, maturation, and migration capacity of DCs. Mice were immunised with PBS, irradiated Hepa 1‐6, or Hepa 1‐6‐mGM‐CSF cells subcutaneously as mentioned before. Twenty‐four hours after immunisation, we discovered an increased infiltration of CD45^+^CD11c^+^MHC‐II^+^ DCs in the skin tissues (Figure 4E). After subcutaneous injection into the hind footpad, irradiated Hepa 1‐6‐mGM‐CSF cells enhanced DC maturation in the draining popliteal LNs, evidenced by elevated expression of co‐stimulatory molecules (CD80, CD86, and CD40, Figure 4F). Meanwhile, vaccination with the Hepa 1‐6‐mGM‐CSF vaccine increased the frequencies of CD11c^+^ cells expressing CCR7 in the popliteal LNs, which is a hallmark of migratory DCs (Figure 4G,H) [35]. Collectively, these data suggest that the Hepa 1‐6‐mGM‐CSF vaccine potently drives DC maturation, cytokine production and migration to lymphoid tissues, all of which are critical steps for efficient antigen presentation and T cell priming.

### 3.5 Irradiation Induces Oxidative Damage to mtDNA and Promotes DC Phagocytosis

**FIGURE 5:**
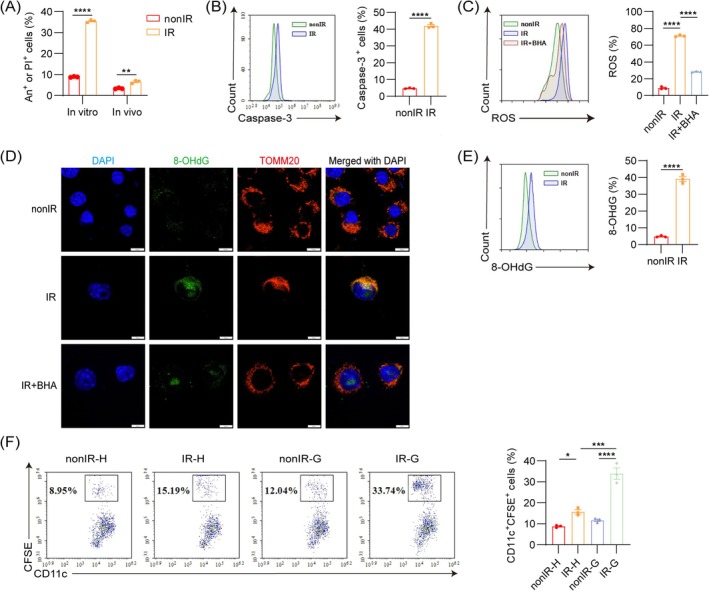
Irradiation induces cell apoptosis, causes oxidative damage to mtDNA and promotes antigen uptake by DCs. (A) Non‐irradiated Hepa 1‐6 cells (nonIR) and irradiated Hepa 1‐6 cells (IR, 100 Gy) were cultured in vitro or injected intraperitoneally into mice. Twenty‐four hours later, FCM was performed to determine the percentages of Annexin V^+^ or PI^+^ cells in the cultured cells (in vitro) or cells in the ascites (in vivo). An, Annexin V. (B) Caspase‐3 expression was detected by FCM. (C) Hepa 1‐6 cells were treated with X‐ray irradiation in the presence of 100 μM BHA (IR + BHA). Intracellular ROS production in Hepa 1‐6 cells was detected using FCM. (D) Immunofluorescence staining with anti‐8‐OHdG antibody (green), TOMM20 antibody (red) and DAPI (cell nuclei, blue) was performed in Hepa 1‐6 cells. Scale bar, 10 μm. (E) 8‐OHdG expression was detected by FCM. (F) BMDCs were cultured with non‐irradiated (nonIR‐H) or irradiated (IR‐H) CFSE‐stained Hepa 1‐6 cells, or non‐irradiated (nonIR‐G) or irradiated (IR‐G) CFSE‐stained Hepa 1‐6‐mGM‐CSF cells for 3 h in vitro. The representative FCM images and quantifications of CD11c^+^CFSE^+^ cells are shown. *n* = 3 per group. Data are shown as mean ± SEM and analysed with (A, B, E) unpaired two‐tailed Student's *t*‐tests, (C, F) one‐way ANOVA test. *p < 0.05, **p < 0.01, ***p < 0.001, ****p < 0.0001.

The results above demonstrate that the tumour cell vaccine loaded with mGM‐CSF exhibits more pronounced anti‐tumour effects compared to irradiated tumour cells alone. Our previous research demonstrated that irradiated tumour cells suppressed tumour growth by activating the STING pathway through the released ox‐mtDNA [20]. In this study, we proposed that the Hepa 1‐6‐mGM‐CSF vaccine exerts its anticancer effects through the synergistic effect of the ox‐mtDNA‐STING pathway and GM‐CSF cytokine production. To test this hypothesis, we first clarified the effects of high‐dose irradiation (100 Gy) on Hepa 1‐6 cells. After 24 h of irradiation, the proportion of PI‐positive or Annexin V‐positive cells significantly increased in both in vitro and in vivo experiments (Figure 5A). To further confirm cell apoptosis induced by X‐ray irradiation, we stained irradiated cells using caspase‐3 antibody, a marker of apoptosis [36]. The results showed that the percentage of caspase‐3‐positive Hepa 1‐6 cells in the irradiated group was significantly higher than that in the non‐irradiated group (Figure 5B).

We also detected intracellular ROS expression of Hepa 1‐6 cells following radiation exposure. Irradiation increased the ROS levels in Hepa 1‐6 cells, while BHA, a ROS scavenger, was shown to significantly decrease ROS expression of irradiated cells (Figure 5C) [37]. The mtDNA is not protected by histones or DNA‐binding proteins, making it more susceptible to oxidative damage than nuclear DNA (nDNA) [38]. 8‐OHdG is formed when ROS attacks DNA molecules, serving as a key biomarker for oxidative DNA damage [39]. We performed immunofluorescence detection of 8‐OHdG and translocase of outer mitochondrial membrane 20 (TOMM20), a mitochondrial marker, in Hepa 1‐6 cells under different treatment conditions [40]. As shown in Figure 5D, radiation‐treated Hepa 1‐6 cells exhibited substantial cytoplasmic expression of 8‐OHdG, which colocalized with TOMM20 signals. Following BHA treatment, cytoplasmic 8‐OHdG levels in these cells decreased significantly (Figure 5D). FCM results also demonstrated increased expression of 8‐OHdG in irradiated cells (Figure 5E). Therefore, radiation exposure induced cell apoptosis and oxidative damage to mtDNA in tumour cells. As described in previous studies, irradiated tumour cells deliver their ox‐mtDNA to the cytoplasm of DCs via cell‐to‐cell contact, where it is recognised by cytoplasmic cGAS of DCs and activates the STING pathway [20, 25]. We then investigated the phagocytic capacity of DCs toward irradiated cells. BMDCs were co‐cultured with irradiated and non‐irradiated CFSE‐labelled cells for 3 h. Results demonstrated that BMDCs engulfed more irradiated cells than non‐irradiated cells (Figure 5F). Moreover, BMDCs engulfed more irradiated Hepa 1‐6‐mGM‐CSF cells than irradiated Hepa 1‐6 cells (Figure 5F). Therefore, we concluded that both irradiation and GM‐CSF were capable of enhancing the phagocytic capacity of DCs.

### 3.6 Hepa 1‐6‐mGM‐CSF Vaccine Enhances DC Activation and Antigen‐Presentation Through Ox‐mtDNA Release and GM‐CSF Production

**FIGURE 6:**
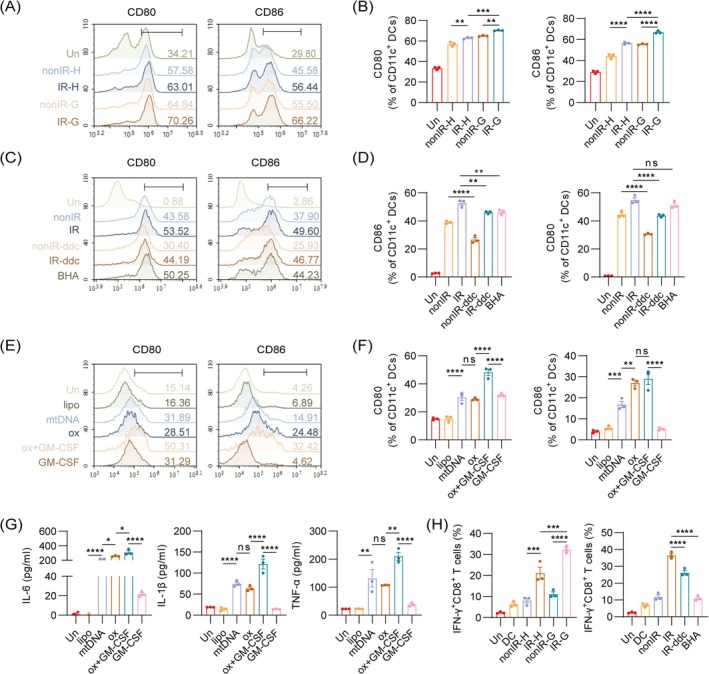
Synergistic effect of ox‐mtDNA and GM‐CSF mediates DC activation by the Hepa 1‐6‐mGM‐CSF vaccine. (A, B) BMDCs (Un) were cultured with non‐irradiated (nonIR‐H) or irradiated (IR‐H) Hepa 1‐6 cells, or non‐irradiated (nonIR‐G) or irradiated (IR‐G) Hepa 1‐6‐mGM‐CSF cells for 24 h. The percentages of CD80^+^ or CD86^+^ cells among CD11c^+^ DCs are shown. (C, D) BMDCs (Un) were stimulated with non‐irradiated (nonIR) or irradiated (IR) Hepa 1‐6‐mGM‐CSF cells, ddC‐treated non‐irradiated (nonIR‐ddC) or irradiated (IR‐ddC) Hepa 1‐6‐mGM‐CSF cells, or BHA‐treated irradiated (BHA) Hepa 1‐6‐mGM‐CSF cells for 24 h. The percentages of CD80^+^ or CD86^+^ cells among CD11c^+^ DCs are shown. (E–G) BMDCs (Un) were stimulated with lipo3000 (lipo), 2 μg/mL mtDNA, 2 μg/mL ox‐mtDNA (ox), ox‐mtDNA plus GM‐CSF (ox + GM‐CSF) or 200 ng/mL GM‐CSF for 24 h. The percentages of CD80^+^ or CD86^+^ cells among CD11c^+^ DCs and the levels of IL‐6, IL‐1β and TNF‐α in the supernatant are shown. (H) Vaccine‐stimulated BMDCs (DC) were co‐cultured with splenic lymphocytes (Un) for 48 h in the presence/absence of ddC/BHA. The proportion of IFN‐γ^+^CD8^+^ T cells was assessed with FCM. *n* = 3 per group. Data are shown as mean ± SEM and analysed with one‐way ANOVA test. *p < 0.05, **p < 0.01, ***p < 0.001, ****p < 0.0001, ns, not statistically significant.

After antigen uptake, DCs up‐regulate the expression of T cell co‐stimulatory molecules (e.g., CD80 and CD86) and cytokines, which drive maturation of DCs, activation of naïve T cells, and subsequent migration of DCs to lymphoid organs. In our study, we have proved that irradiation and GM‐CSF jointly promote DC phagocytosis (Figure 5F). We next stimulated BMDCs with non‐irradiated or irradiated Hepa 1‐6 cells, or non‐irradiated or irradiated Hepa 1‐6‐mGM‐CSF cells for 24 h to investigate their maturation status. FCM analysis revealed that BMDCs exhibited higher expression of CD80 and CD86 in the irradiated cells group compared to the non‐irradiated cells group (Figure 6A,B). Besides, irradiated Hepa 1‐6‐mGM‐CSF cells promoted BMDC maturation more effectively than irradiated Hepa 1‐6 cells (Figure 6A,B). To determine whether the ability of the Hepa 1‐6‐mGM‐CSF vaccine to activate DCs is associated with ox‐mtDNA, Hepa 1‐6‐mGM‐CSF cells were treated with dideoxycytidine (ddC), an inhibitor of mtDNA polymerase γ, thereby reducing mtDNA copy numbers while leaving nDNA unaffected [41]. After 6 days of treatment, we measured the ratio of mtDNA to nDNA in the ddC‐treated Hepa 1‐6‐mGM‐CSF cells and found that mtDNA was effectively depleted (Figure S3D). BMDCs were co‐cultured with ddC‐treated or BHA‐treated Hepa 1‐6‐mGM‐CSF cells for 24 h. The results indicated that depletion of mtDNA or reduction of ROS production in Hepa 1‐6‐mGM‐CSF cells markedly inhibited the maturation of BMDCs (Figure 6C,D).

To further explore the functions of ox‐mtDNA and GM‐CSF in Hepa 1‐6‐mGM‐CSF vaccine‐induced DC activation, we isolated mtDNA and ox‐mtDNA from non‐irradiated and irradiated Hepa 1‐6 cells, respectively. Then, BMDCs were stimulated with mtDNA, ox‐mtDNA, GM‐CSF, or ox‐mtDNA plus GM‐CSF. GM‐CSF alone significantly up‐regulated CD80 expression (31.97% ± 0.68%) and induced a modest increase in CD86 (5.17% ± 0.37%), whereas incubation with mtDNA induced only a modest upregulation of CD80 and CD86. In contrast, ox‐mtDNA stimulation resulted in comparable CD80 (29.02% ± 0.59%) expression but markedly higher CD86 (27.04% ± 1.31%) expression than mtDNA, indicating that oxidative modification of mtDNA enhances its immunostimulatory potential. Notably, combined stimulation with GM‐CSF and ox‐mtDNA elicited the highest levels of CD80 (48.21% ± 2.30%) and CD86 (29.13% ± 2.78%) expression in BMDCs (Figure 6E,F). In line with these findings, stimulated BMDCs exhibited a similar trend in pro‐inflammatory cytokine production, with ox‐mtDNA and GM‐CSF co‐stimulation eliciting the highest levels of IL‐6 (310.04 ± 22.57 pg/mL), IL‐1β (121.74 ± 11.63 pg/mL) and TNF‐α (209.82 ± 14.46 pg/mL) compared to ox‐mtDNA (257.37 ± 5.75, 63.62 ± 3.80, 107.08 ± 0.58 pg/mL) and GM‐CSF (21.71 ± 1.57, 14.28 ± 0.62, 37.44 ± 3.45 pg/mL) group (Figure 6G).

Additionally, to evaluate the capacity of BMDCs to induce T cell activation, pre‐stimulated BMDCs were co‐cultured with splenic lymphocytes isolated from C57BL/6 mice. CD8^+^ T cells can secrete large amounts of effector molecules, such as interferon‐γ (IFN‐γ), to kill tumour cells [42]. The results showed that the percentage of IFN‐γ^+^CD8^+^ T cells was higher in the irradiated cells group than the non‐irradiated cells group, with the highest level observed in the Hepa 1‐6‐mGM‐CSF vaccine group (Figure 6H). Moreover, depleting mtDNA and ROS levels in irradiated Hepa 1‐6‐mGM‐CSF cells significantly suppressed the proportion of IFN‐γ^+^CD8^+^ T cells (Figure 6H). Collectively, these results indicated that the Hepa 1‐6‐mGM‐CSF vaccine promotes the activation and antigen‐presentation capacity of DCs by releasing ox‐mtDNA and GM‐CSF simultaneously.

### 3.7 Ox‐mtDNA Mediates Anti‐tumour Immunity by Activating the STING Pathway in DCs

**FIGURE 7:**
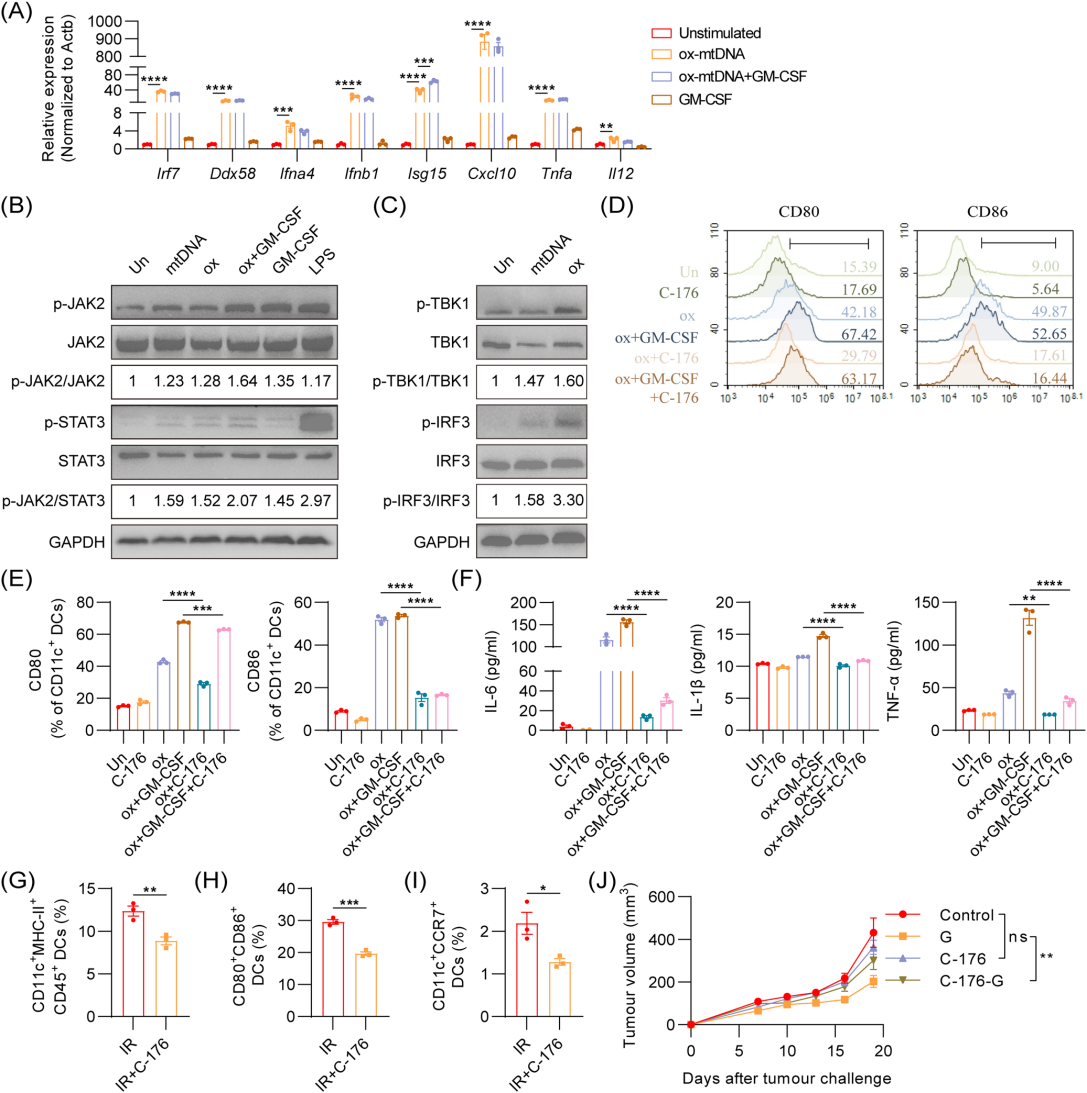
Ox‐mtDNA‐mediated anti‐tumour immunity depends on activation of the STING pathway in DCs. (A) BMDCs were stimulated by ox‐mtDNA, ox‐mtDNA plus GM‐CSF and GM‐CSF for 3 h. Relative mRNA levels of the indicated genes in BMDCs were measured by RT‐qPCR. (B, C) BMDCs (Un) were stimulated by mtDNA, ox‐mtDNA (ox), ox‐mtDNA plus GM‐CSF (ox + GM‐CSF), GM‐CSF or 1 μg/mL LPS for 3 h. Western blot was used to detect the expression levels of the indicated proteins. (D–F) BMDCs (Un) were treated with 0.2 μM C‐176 when stimulated with ox‐mtDNA or ox‐mtDNA plus GM‐CSF. The percentages of CD80^+^ or CD86^+^ cells among CD11c^+^ DCs and the levels of IL‐6, IL‐1β and TNF‐α in the supernatant are shown. (G–I) Mice were intraperitoneally administered C‐176 and subsequently injected with irradiated Hepa 1‐6‐mGM‐CSF cells or PBS subcutaneously in the dorsal flank or hind footpad. The percentages of DCs (CD45^+^CD11c^+^MHC‐II^+^, G) in the skin and mature (CD80^+^CD86^+^, G) or migrated (CD11c^+^CCR7^+^, H) DCs in popliteal LNs were assessed with FCM. (J) Mice were subcutaneously administered with PBS or 5 × 10^5^ irradiated Hepa 1‐6‐mGM‐CSF cells (G), along with daily intraperitoneal injections of 13.4 mg/kg C‐176 (C‐176/C‐176‐G) or the corresponding solvent. On day 7 post‐immunisation, 5 × 10^6^ Hepa 1‐6 cells were subcutaneously inoculated into the mice. Tumour volume was monitored after injection. (A, D–I) *n* = 3 per group. (J) *n* = 5 per group. Data are shown as mean ± SEM and analysed with (A, E, F) one‐way ANOVA test, (G–I) unpaired two‐tailed Student's *t*‐tests and (J) two‐way ANOVA test. *p < 0.05, **p < 0.01, ***p < 0.001, ****p < 0.0001, ns, not statistically significant.

Given that cytosolic mtDNA is a potent activator of the STING pathway, we next investigated the activation of the cGAS‐STING pathway in DCs [43, 44]. After recognising and binding to DNA, cGAS activates STING, which in turn stimulates TANK‐binding kinase 1 (TBK1) and interferon regulatory factor 3 (IRF3) [45]. Phosphorylated IRF3 translocates to the nucleus to induce expression of type I interferons, interferon‐stimulated genes (ISGs) and other genes [45]. Critically, phosphorylated TBK1 and IRF3 are well‐established, downstream hallmarks of STING pathway activation [20, 46]. In BMDCs stimulated with ox‐mtDNA, elevated mRNA transcription levels of the cGAS‐STING pathway and downstream type I interferon responses were confirmed (Figure 7A). The results also indicated that GM‐CSF exhibited a limited effect on the STING pathway, except for increased *Isg15* gene expression (Figure 7A). The GM‐CSF‐induced higher expression of *Isg15* gene may be due to the activation of the JAK–STAT pathway in DCs, as ISG15 is a key protein involved in regulating JAK/STAT signalling [47]. Consistent with this speculation, Western blot results revealed that GM‐CSF significantly promoted the expression of phosphorylated JAK2 (p‐JAK2) and phosphorylated STAT3 (p‐STAT3) (Figure 7B) in BMDCs. Meanwhile, the expression levels of phosphorylated TBK1 (p‐TBK1) and phosphorylated IRF3 (p‐IRF3), key proteins in the cGAS‐STING pathway, were greatly elevated in the ox‐mtDNA group (Figure 7C). These results confirmed that ox‐mtDNA promotes STING pathway activation in BMDCs.

To prove the involvement of the STING pathway, we treated BMDCs with a selective inhibitor of STING, C‐176, when stimulating BMDCs with ox‐mtDNA or ox‐mtDNA plus GM‐CSF. FCM and ELISA analyses demonstrated that C‐176 significantly decreased the expression of CD80 and CD86 and inhibited cytokine production (IL‐6, IL‐1β and TNF‐α) in BMDCs following stimulation with ox‐mtDNA or ox‐mtDNA plus GM‐CSF (Figure 7D–F). In vivo experiments demonstrated that C‐176 treatment lowered the infiltration of DCs (CD45^+^CD11c^+^MHC‐II^+^, Figure 7G) in the skin and inhibited their maturation (CD80 and CD86 expression, Figure 7H) and migration (CCR7, Figure 7I) to popliteal LNs.

**FIGURE 8:**
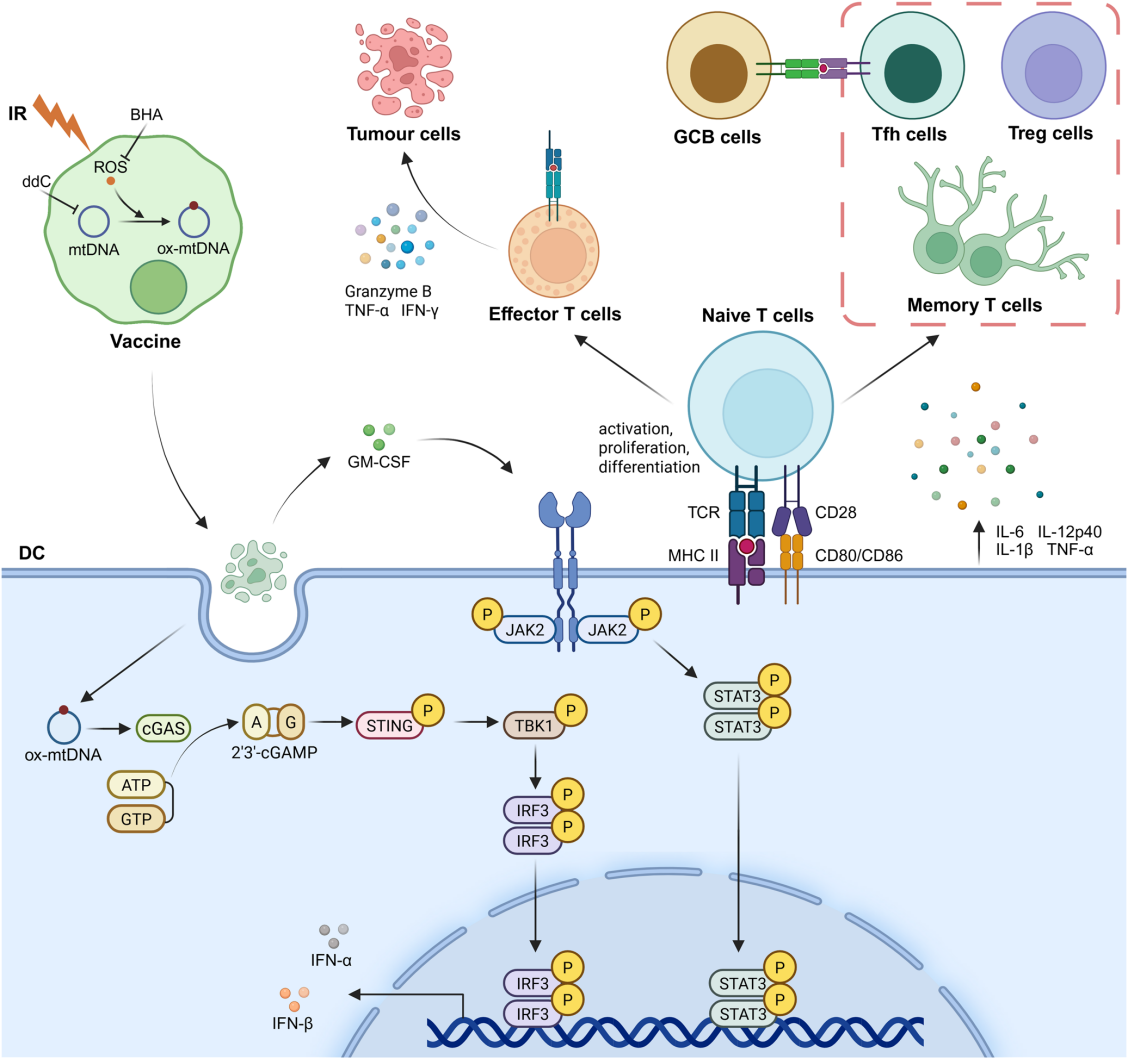
Schematic summary of the underlying mechanisms of the prophylactic efficacy of the Hepa 1‐6‐mGM‐CSF vaccine.

Finally, we determined the impact of the STING signalling on the protective effect of the Hepa 1‐6‐mGM‐CSF vaccine. After immunisation with irradiated Hepa 1‐6‐mGM‐CSF cells, mice were implanted with live Hepa 1‐6 cells subcutaneously while receiving daily intraperitoneal injections of C‐176. We found that inhibition of the STING pathway significantly impaired the anti‐tumour effect of the cell vaccine (Figure 7J). Therefore, we conclude that the Hepa 1‐6‐mGM‐CSF vaccine promotes the phagocytic capacity, maturation, migration and antigen‐presentation of DCs through the synergistic interaction of the ox‐mtDNA‐mediated STING pathway activation and GM‐CSF, which contribute to specific and long‐lasting anti‐tumour immunity (Figure 8).

## 4 Discussion

To date, liver cancer remains one of the leading causes of cancer‐related mortality globally, posing a formidable challenge to healthcare systems worldwide [1]. Given the limitations of traditional treatments and the growing prominence of immunotherapy, tumour vaccines may be a promising new approach for treating liver cancer. However, the immune responses induced by irradiated tumour cells alone are relatively weak. Therefore, we incorporated GM‐CSF into the vaccine formulation, a factor crucial for DC function that enhances therapeutic efficacy in multiple tumour models [48, 49, 50]. In this study, we established a novel vaccine by engineering the Hepa 1‐6 cell line to overexpress murine GM‐CSF, enabling high‐level secretion of GM‐CSF. Our findings demonstrated that irradiated Hepa 1‐6 cells alone elicited robust prophylactic anti‐tumour immunity, effectively inhibiting primary tumour growth. Furthermore, the GM‐CSF‐loaded Hepa 1‐6 cell vaccine induced stronger anti‐tumour effects, providing long‐lasting immune protection and preventing tumour recurrence, which was consistent with previous research [51]. Moreover, we evaluated the safety of the Hepa 1‐6‐GM‐CSF vaccine. The vaccine showed no adverse effects in mice, with no signs of hair loss, weight loss, or behavioural abnormalities. Histopathological and serum biochemical analyses of major organs revealed no significant changes, confirming its excellent safety profile.

To unveil the underlying mechanisms of the therapeutic efficacy of the tumour cell vaccine, we examined the immune responses elicited in the immunised mice. As expected, prophylactic immunisation with the Hepa 1‐6‐mGM‐CSF vaccine significantly increased the frequency of mature DCs, enhanced T cell immune responses and stimulated the humoral arm of the adaptive immune system. Meanwhile, we observed a significant reduction in Tregs, immune‐inhibitory cells that can promote tumour development and progression [52]. Beyond that, we found that the Hepa 1‐6‐mGM‐CSF vaccine profoundly reshaped the TME through increasing the infiltration of mature DCs and M1 macrophages and reducing the local recruitment of the immunosuppressive MDSCs [53]. Interestingly, unlike in non‐tumour bearing mice where the vaccine primarily promoted the elevation of T_CM_ cells, a primary increase in T_EM_ cells was observed in tumour‐bearing mice that received the Hepa 1‐6‐mGM‐CSF vaccine (Figures 2E,F and 3H,I). The differentiation of memory T cells is influenced by numerous factors, including antigen concentration, strength or duration of TCR signalling, CD4^+^ T cell help, co‐stimulatory or adhesion molecules expression and the cytokines in the microenvironment [54]. It has been shown that weaker stimulation is more conducive to the generation of T_CM_ [54]. Another study proposes a linear differentiation model, in which naïve T cells first differentiate into T_CM_ cells, and subsequently differentiate into T_EM_ cells according to the intensity and duration of TCR stimulation, as well as the presence or absence of polarising cytokines [55]. Therefore, we hypothesise that the increase in T_EM_ cells instead of T_CM_ cells in the tumour‐bearing mice may be attributable to the intensity of antigen stimulation. It also explains why irradiated Hepa 1‐6 cells alone demonstrated comparable tumour growth inhibition to Hepa 1‐6‐GM‐CSF cells in the prophylactic experiment but failed to suppress tumour recurrence in the rechallenge experiment. This may be attributed to the weaker memory immunity induced by irradiated Hepa 1‐6 cells, which led to a shorter and ultimately diminished anti‐tumour immune response.

DCs are crucial for the initiation of T cell‐mediated tumour immunity. Immature DCs recognise pathogen‐associated molecular patterns (PAMPs) or DAMPs through their surface pattern recognition receptors (PRRs), thereby inducing their maturation [56]. Mature DCs up‐regulate MHC molecules and co‐stimulatory molecules (such as CD80, CD86 and CD40), and produce pro‐inflammatory cytokines (such as IL‐6, IL‐1β, IL‐12 and TNF‐α) [57, 58]. Additionally, mature DCs up‐regulate the expression of chemokine receptor CCR7, migrate to secondary lymphoid organs and present antigens to helper T cells or effector T cells, thereby triggering specific cytotoxic T lymphocyte (CTL) responses [35]. A recent study reported that a GM‐CSF‐producing cellular cancer vaccine formulated with a STING agonist exhibited significant anti‐tumour responses with increased activation of DCs, and infiltration of antigen‐specific CD8^+^ T cells [59]. Similarly, we demonstrated that the Hepa 1‐6‐mGM‐CSF vaccine effectively promoted DC recruitment, maturation and migration into the draining LNs. Consistent with previous research, irradiated tumour cells alone could induce DC activation and modest anti‐tumour responses [20]. However, loading GM‐CSF into the cell‐based vaccine exhibited more pronounced immunostimulatory effects. Therefore, it can be speculated that the Hepa 1‐6‐mGM‐CSF vaccine's tumour‐suppressing function is mediated by the synergistic action of irradiated tumour cells and the simultaneously produced GM‐CSF.

Radiation exposure significantly increased ROS levels within tumour cells. Due to differences in the structures of mtDNA and nuclear DNA, mtDNA is more susceptible to ROS damage [38]. This is confirmed by the colocalization of 8‐OHdG, a marker of oxidative DNA damage, and TOMM20, a mitochondrial marker, in the immunofluorescence images (Figure 5D). Previous studies have confirmed this observation by showing that elevated levels of ROS induce oxidative damage to mtDNA in human hepatocellular carcinoma cell lines, such as Huh7 [60]. Compared to unmodified mtDNA, ox‐mtDNA has been proven to be a more potent agonist of the DNA‐sensing STING pathway [61]. The PCR and Western blot results also revealed that ox‐mtDNA activated the STING pathway more effectively in DCs, which was independent of GM‐CSF. A previous study demonstrated that oxidised DNA binds to cGAS in mouse myeloid DCs to form c[G(2′5′)pA(3′5′)p] complex, directly promoting the STING pathway activation and downstream type I IFN production [61]. Another research reported that irradiated tumour cells were efficiently phagocytosed by DCs, with ox‐mtDNA entering the DC cytoplasm where it activated the cGAS‐STING pathway [20]. Therefore, irradiation‐induced ox‐mtDNA in tumour cells is crucial for activating the STING pathway in DCs.

GM‐CSF also exerts a profound effect on DC function, including stimulating the activation and migration of DCs [24]. At the cellular level, we demonstrated that ox‐mtDNA‐induced STING pathway activation and GM‐CSF jointly promoted antigen uptake capacity, DC maturation and subsequent activation of effector CD8^+^ T cells. However, at the molecular level, we observed an intriguing phenomenon that ox‐mtDNA preferentially up‐regulated CD86 expression (Figure 6E,F). It was reported that native mtDNA significantly up‐regulated the expression of CD86 in human plasmacytoid DCs (pDCs), while ox‐mtDNA triggered a stronger increase of CD86 expression, which depends on toll‐like receptor 9 (TLR9) [62]. Although PCR data indicated that GM‐CSF was unable to activate the STING pathway, it is capable of stimulating multiple other signalling cascades, including JAK/STAT, PI3K, MAPK and NF‐κB signalling pathways [63]. Consistent with this, our PCR results demonstrated up‐regulated expression of *Isg15* gene, a canonical interferon‐stimulated gene, which is crucial for regulating JAK/STAT signalling (Figure 7A) [47]. This finding was further corroborated by Western blot results showing GM‐CSF specifically activated the JAK2/STAT3 signalling axis in DCs (Figure 7B). GM‐CSF itself does not activate STING pathway, while crosstalk exists between these two pathways. Activation of the STING pathway stimulates production of type I interferons (IFN‐I) which, upon binding to their receptors IFNAR1/IFNAR2, leads to phosphorylation of JAK kinases including TYK2 and JAK1/2, thereby activating downstream signalling pathways [64]. A study in an intestinal ischemia–reperfusion (IIR) model demonstrated that STING up‐regulation enhanced IFN‐γ production, which activated the JAK2/STAT3 pathway and led to M1 macrophage polarisation [65]. Our results also demonstrated the synergic effects of ox‐mtDNA and GM‐CSF on enhancing DCs maturation, antigen‐presentation, infiltration and migration. These findings underscore that incorporating GM‐CSF into tumour cell vaccine is not only beneficial but also essential for enhancing innate immune activation and promoting more effective adaptive responses, thereby maximising anti‐tumour immunity. Meanwhile, our data revealed that the STING pathway is indispensable for anti‐tumour efficacy of irradiated Hepa 1‐6‐mGM‐CSF vaccine, which was consistent with prior evidence (Figure 7J) [20]. Given the critical role of STING pathway in anti‐tumour immunity, these mechanistic insights directly inform the rational incorporation of STING agonists into next‐generation tumour vaccine platforms [66].

Despite the promising results, this study has several limitations. First, the application of subcutaneous tumour models provides a valuable platform for exploring the preliminary efficacy and mechanistic exploration of the constructed vaccine. Future studies integrating in situ models or humanised mice are needed to further elucidate the complexity of the liver microenvironment. Second, the prophylactic vaccination and tumour rechallenge experiment effectively established the foundation for vaccine‐induced immune memory and long‐lasting protection. However, therapeutic vaccination models in subsequent studies are expected to more comprehensively reflect clinical scenarios. Third, although the current findings implicate the STING pathway and GM‐CSF as key mechanisms, further exploration of complementary pathways may help elucidate the vaccine's biological activity. Finally, as mouse models serve as crucial initial steps, subsequent validation in humanised systems is essential to provide a foundation for future clinical development.

## 5 Conclusion

In this study, we developed an mGM‐CSF‐loaded liver cancer cell‐based vaccine that markedly suppressed tumour growth and conferred robust protection against tumour rechallenge with the same hepatocellular carcinoma cells. Immunisation with this vaccine was sufficient to induce potent activation of DCs and elicit tumour‐specific T cell responses both in vitro and in vivo. Mechanistically, radiation‐induced oxidative damage to mtDNA in cancer cells activated the cGAS‐STING signalling pathway in DCs following phagocytosis. This innate immune activation acted synergistically with overexpressed mGM‐CSF to promote DC maturation and migration to draining lymph nodes, thereby effectively initiating adaptive anti‐tumour immunity.

## Author Contributions

Zhiruo Song, Yujie Jiang and Yu Zhang contributed equally to this work. Xiawei Wei, Aqu Alu and Xuemei He developed the project and offered the main direction of this study. Aiping Tong provided the plasmid carrying the target gene. Zhiruo Song, Yujie Jiang and Aqu Alu conducted vaccination experiments on mice. Zhiruo Song, Aqu Alu, Yujie Jiang, Yu Zhang, Danyi Ao, Chunjun Ye, Xiya Huang, Yingqiong Zhou, Hanle Yang, Ruolan Xia and Jiayuan Ai collected lymph nodes, spleens, tumour samples and skin tissues and performed flow cytometry. Yu Zhang collected serum for biochemical analysis. Zhiruo Song, Aqu Alu, Yujie Jiang, Yu Zhang, Dandan Wan and Danyi Ao participated in data analysis. Xiawei Wei, Zhiruo Song, Dandan Wan, Aqu Alu and Xuemei He wrote the manuscript. All authors have revised and agreed to the submitted version of the manuscript.

## Funding

This work was supported by the National Key Research and Development Program of China, 2024YFC2310700; 1·3·5 project for disciplines of excellence‐Clinical Research Fund, West China Hospital, Sichuan University, ZYGD23038; National Natural Science Foundation of China Young Student Basic Research Program, 823B2065; National Natural Science Foundation of China, 82403281; Natural Science Foundation of Sichuan Province, 2024NSFSC1199; Post‐Doctor Research Project, West China Hospital, Sichuan University, 2023HXBH046.

## Ethics Statement

Sichuan University's Institutional Animal Care and Use Committee gave the approval for all animal studies.

## Conflicts of Interest

The authors declare no conflicts of interest.

## Supporting information


**Table S1:** Primers used in qPCR and RT‐qPCR.
**Table S2:** The information of antibodies.
**Figure S1:** The gating method for T cells in spleen and DCs in lymph node. (A) The representative FCM images and method of gating proliferative CD4^+^ and CD8^+^ T cells (CFSE^low^) in splenic lymphocytes of immunised mice after being stimulated by irradiated Hepa 1‐6 cells for 72 h. (B) The representative FCM images and method of gating T_EM_ in spleens of immunised mice challenged with 5 × 10^6^ Hepa 1‐6 cells. (C) The representative FCM images and method of gating CD11c^+^CCR7^+^ cells in popliteal LNs are shown. FCM, flow cytometry; LN, lymph node.
**Figure S2:** The gating method for BMDCs. (A) BMDCs were cultured with non‐irradiated or irradiated CFSE‐stained cells for 3 h in vitro. The representative FCM images and method of gating CD11c^+^CFSE^+^ cells are shown. (B) BMDCs were cultured with non‐irradiated or irradiated cells for 24 h in vitro. The representative FCM images and method of gating CD80^+^ or CD86^+^ cells are shown.
**Figure S3:** Assessment of ddC‐mediated mitochondrial depletion in cells and vaccine safety. (A) The ratio of mtDNA to nuclear DNA was quantified by real‐time PCR, with untreated Hepa1‐6 cells serving as the control. (B) On days 0, 14 and 28, mice were administered subcutaneously with PBS, 5 × 10^5^ irradiated Hepa 1‐6 cells, or 5 × 10^5^ irradiated Hepa 1‐6‐mGM‐CSF cells. Body weight was measured on day 1 post‐immunisation. (C, D) On day 7 following the third immunisation, serum and major organs (heart, liver, spleen, lung and kidney) were collected. (C) The biochemical parameters and (D) hematoxylin and eosin (H&E) staining of major organs were performed. (A) *n* = 3 per group. (B–D) *n* = 5 per group. Data are shown as mean ± SEM. ****p < 0.0001, ns, not statistically significant.

## Data Availability

All the data in this research are available by requesting the corresponding author.
