# Supplementary material for: Irradiated Liver Cancer Cell Vaccine Transfected With GM‐CSF Induces Specific and Long‐Lasting Anti‐tumour Immunity Through the Synergistic Effect of Oxidised mtDNA and GM‐CSF
Source: Cell Prolif. 2026 Apr 8;59(8):e70198. doi: 10.1111/cpr.70198 (PMC13326035; doi:10.1111/cpr.70198)
Supplement: Supplementary file 1 [file CPR-59-e70198-s001.docx]

**Supplementary Materials**

**Irradiated Liver Cancer Cell Vaccine Transfected With GM‐CSF Induces Specific and Long‐Lasting Anti‐tumour Immunity Through the Synergistic Effect of Oxidised mtDNA and GM‐CSF**

Zhiruo Song^1^, Yujie Jiang^1^, Yu Zhang^1^, Danyi Ao^1^, Chunjun Ye^1^, Xiya Huang^1^, Yingqiong Zhou^1^, Hanle Yang^1^, Ruolan Xia^1^, Jiayuan Ai^1^, Dandan Wan^1^, Aiping Tong^1^, Yuquan Wei^1^, Xuemei He^1, ⁎^, Aqu Alu^1, ⁎^, Xiawei Wei^1, ⁎^

^1^Laboratory of Aging Research and Cancer Drug Target, State Key Laboratory of Biotherapy and Cancer Center, National Clinical Research Center for Geriatrics, West China Hospital, Sichuan University, Chengdu 610041, China.

^⁎^Corresponding authors: Xuemei He ([hexuemei1116@126.com](mailto:hexuemei1116@126.com)), Aqu Alu ([aluaqu@qq.com](mailto:aluaqu@qq.com)) and Xiawei Wei (xiaweiwei@scu.edu.cn)

**Includes:**

**Supplementary Table 1 and 2**

**Supplementary Figure 1-3**

**Tables**

**Supplementary Table 1. Primers used in qPCR and RT-qPCR**

| Gene name | Forward sequences  Reverse sequences |
| --- | --- |
| β-actin | AGAGGGAAATCGTGCGTGAC  CAATAGTGATGACCTGGCCGT |
| mGM-CSF | GCTCTAGAAGATCACCGGCGAAGGA  TATGCGGCCGCTTCCTCATTTTTGGCC |
| mtDNA (cytochrome c oxidase I) | GCCCCAGATATAGCATTCCC  GTTCATCCTGTTCCTGCTCC |
| nDNA (18S ribosomal RNA) | TAGAGGGACAAGTGGCGTTC  CGCTGAGCCAGTCAGTGT |
| *mIrf7* | CAATTCAGGGGATCCAGTTG  AGCATTGCTGAGGCTCACTT |
| *mDdx58* | GAGTACCACTTAAAGCCAGAG  AATCCATTTCTTCAGAGCATCC |
| *mIfna4* | CTTTCCTCATGATCCTGGTAATGAT  AATCCAAAATCCTTCCTGTCCTTC |
| *mIfnb1* | CCCTATGGAGATGACGGAGA  CCCAGTGCTGGAGAAATTGT |
| *mIsg15* | CTAGAGCTAGAGCCTGCAG  AGTTAGTCACGGACACCAG |
| *mCxcl10* | CCAAGTGCTGCCGTCATTTTC  GGCTCGCAGGGATGATTTCAA |
| *mTnfa* | CATCTTCTCAAAATTCGAGTGACAA  CCAGCTGCTCCTCCACTTG |
| *mIl12* | CCATTGAACTGGCGTTGGAAG  ACTTGAGGGAGAAGTAGGAATGG |

**Supplementary Table 2. The information of antibodies.**

| **Antibody** | **Supplier** | **Cat. No.** | **Application** | **Dilution** |
| --- | --- | --- | --- | --- |
| PerCP-Cy5.5 anti-mouse CD45 antibody | Biolegend | 103132 | FCM | 1:100 |
| APC anti-mouse CD11c antibody | Biolegend | 117310 | FCM | 1:100 |
| PE-Cy7 anti-mouse MHC-Ⅱ antibody | Biolegend | 107630 | FCM | 1:100 |
| PE anti-mouse CD80 antibody | Biolegend | 104708 | FCM | 1:100 |
| FITC anti-mouse CD86 antibody | Biolegend | 105006 | FCM | 1:100 |
| PerCP-Cy5.5 anti-mouse CD3 antibody | Biolegend | 100218 | FCM | 1:100 |
| APC anti-mouse CD4 antibody | Biolegend | 100412 | FCM | 1:100 |
| BV510 anti-mouse CD8a antibody | Biolegend | 100752 | FCM | 1:100 |
| PE-Cy7 anti-mouse CD69 antibody | Biolegend | 104512 | FCM | 1:100 |
| FITC anti-mouse CD4 antibody | Biolegend | 100406 | FCM | 1:100 |
| APC anti-mouse CD8a antibody | Biolegend | 100712 | FCM | 1:100 |
| BV510 anti-mouse/human CD44 antibody | Biolegend | 103044 | FCM | 1:100 |
| PE-Cy7 anti-human/mouse Granzyme B recombinant antibody | Biolegend | 372214 | FCM | 1:100 |
| PE anti-mouse TNF-α antibody | Biolegend | 506306 | FCM | 1:100 |
| PE anti-mouse CD44 antibody | Biolegend | 103008 | FCM | 1:100 |
| BV421 anti-mouse CD62L antibody | Biolegend | 104436 | FCM | 1:100 |
| BV510 anti-mouse CD4 antibody | Biolegend | 100449 | FCM | 1:100 |
| BV421 anti-mouse CD279 (PD-1) antibody | Biolegend | 135218 | FCM | 1:100 |
| FITC anti-mouse CD185 (CXCR5) antibody | Biolegend | 145520 | FCM | 1:100 |
| PE anti-mouse CD19 antibody | Biolegend | 152408 | FCM | 1:100 |
| BV421 anti-mouse/human GL7 antigen antibody | Biolegend | 144614 | FCM | 1:100 |
| FITC anti-mouse CD95 (Fas) antibody | Biolegend | 152606 | FCM | 1:100 |
| APC anti-mouse CD25 antibody | Biolegend | 102012 | FCM | 1:100 |
| PE anti-mouse/rat/human Foxp3 antibody | Biolegend | 320008 | FCM | 1:100 |
| PE anti-mouse CD3 antibody | Biolegend | 100206 | FCM | 1:100 |
| BV421 anti-mouse CD4 antibody | Biolegend | 100563 | FCM | 1:100 |
| BV421 anti-mouse/human CD11b antibody | Biolegend | 101251 | FCM | 1:100 |
| PE anti-mouse F4/80 antibody | Biolegend | 123110 | FCM | 1:100 |
| PE anti-mouse CD45 antibody | Biolegend | 103106 | FCM | 1:100 |
| PE-Cy7 anti-mouse/human CD11b antibody | Biolegend | 101216 | FCM | 1:100 |
| PerCP-Cy5.5 anti-mouse Ly-6G/Ly-6C (Gr-1) antibody | Biolegend | 108428 | FCM | 1:100 |
| PE anti-mouse CD8a antibody | Biolegend | 100708 | FCM | 1:100 |
| BV510 anti-mouse CD11c antibody | Biolegend | 117338 | FCM | 1:100 |
| APC anti-mouse CD40 antibody | Biolegend | 124612 | FCM | 1:100 |
| PE anti-mouse CD45 antibody | Biolegend | 103106 | FCM | 1:100 |
| PE anti-mouse CD80 antibody | Biolegend | 104708 | FCM | 1:100 |
| BV421 anti-mouse CD40 antibody | Biolegend | 124641 | FCM | 1:100 |
| PerCP-Cy5.5 anti-mouse CD197 (CCR7) antibody | Biolegend | 120116 | FCM | 1:100 |
| PE-Cy7 anti-mouse CD11c antibody | Biolegend | 117318 | FCM | 1:100 |
| APC anti-mouse CD80 antibody | Biolegend | 104714 | FCM | 1:100 |
| FITC anti-mouse CD8a antibody | Biolegend | 100706 | FCM | 1:100 |
| Anti-caspase-3 antibody | Cell Signaling Technology | 9664 | FCM | 1:400 |
| Anti-8-OHdG antibody | Abcam | Ab62623 | FCM and IF | 1:500 |
| Anti-TOMM20 antibody | Abcam | Ab186734 | IF | 1:250 |
| Anti-phospho-TBK1/NAK (Ser172) rabbit antibody | Cell Signaling Technology | 5483 | WB | 1:1000 |
| Anti-TBK1/NAK rabbit antibody | Cell Signaling Technology | 3013 | WB | 1:1000 |
| Anti-phospho-IRF-3 (Ser396) rabbit antibody | Cell Signaling Technology | 4947 | WB | 1:1000 |
| Anti-IRF3 antibody rabbit antibody | Cell Signaling Technology | 4302 | WB | 1:1000 |
| Anti-phospho-Jak2 (Tyr1007/1008) rabbit antibody | Cell Signaling Technology | 3771 | WB | 1:1000 |
| Anti-Jak2 rabbit antibody | Cell Signaling Technology | 3230 | WB | 1:1000 |
| Anti-phospho-Stat3 (Tyr705) rabbit antibody | Cell Signaling Technology | 9145 | WB | 1:1000 |
| Anti-Stat3 mouse antibody | Cell Signaling Technology | 9139 | WB | 1:1000 |
| Anti-GAPDH rabbit antibody | HUABIO | ET1601-4 | WB | 1:50000 |

Abbreviations: FCM, flow cytometry; IF, immunofluorescence; WB, western blot

**Figures**

**Supplementary Figure 1-3**

**Supplementary Figure 1 The gating method for T cells in spleen and DCs in lymph node.** (A) The representative FCM images and method of gating proliferative CD4^+^ and CD8^+^ T cells (CFSE^low^) in splenic lymphocytes of immunised mice after being stimulated by irradiated Hepa 1-6 cells for 72 h. (B) The representative FCM images and method of gating T_EM_ in spleens of immunised mice challenged with 5 × 10^6^ Hepa 1-6 cells. (C) The representative FCM images and method of gating CD11c^+^CCR7^+^ cells in popliteal LNs are shown. Abbreviations: FCM, flow cytometry; LN, lymph node.

**Supplementary Figure 2 The gating method for BMDCs.** (A) BMDCs were cultured with nonirradiated or irradiated CFSE-stained cells for 3 h in vitro. The representative FCM images and method of gating CD11c^+^CFSE^+^ cells are shown. (B) BMDCs were cultured with nonirradiated or irradiated cells for 24 h in vitro. The representative FCM images and method of gating CD80^+^ or CD86^+^ cells are shown.

**Supplementary Figure 3** **Assessment of ddC-mediated mitochondrial depletion in cells and vaccine safety.** (A) The ratio of mtDNA to nuclear DNA was quantified by real-time PCR, with untreated Hepa 1-6 cells serving as the control. (B) On days 0, 14, and 28, mice were administered subcutaneously with PBS, 5 × 10^5^ irradiated Hepa 1-6 cells, or 5 × 10^5^ irradiated Hepa 1-6-mGM-CSF cells. Body weight was measured on day 1 post-immunisation. (C, D) On day 7 following the third immunisation, serum and major organs (heart, liver, spleen, lung, and kidney) were collected. (C) The biochemical parameters and (D) hematoxylin and eosin (H&E) staining of major organs were performed. (A) n = 3 per group. (B-D) n = 5 per group. Data are shown as mean ± SEM. **** p < 0.0001, ns: not statistically significant.
