# Supplementary material for: Irradiated Liver Cancer Cell Vaccine Transfected With GM‐CSF Induces Specific and Long‐Lasting Anti‐tumour Immunity Through the Synergistic Effect of Oxidised mtDNA and GM‐CSF
Source: Cell Prolif. 2026 Apr 8:e70198. Online ahead of print. doi: 10.1111/cpr.70198 (PMC13326035; doi:10.1111/cpr.70198)
Supplement: Supplementary file 1 — Table S1: Primers used in qPCR and RT‐qPCR. Table S2: The information of antibodies. Figure S1: The gating method for T cells in spleen and DCs in lymph node. (A) The representative FCM images and method of gating proliferative CD4+ and CD8+ T cells (CFSElow) in splenic lymphocytes of immunised mice after being stimulated by irradiated Hepa 1‐6 cells for 72 h. (B) The representative FCM images and method of gating TEM in spleens of immunised mice challenged with 5 × 10^6^ Hepa 1‐6 cells. (C) The representative FCM images and method of gating CD11c+CCR7+ cells in popliteal LNs are shown. FCM, flow cytometry; LN, lymph node. Figure S2: The gating method for BMDCs. (A) BMDCs were cultured with non‐irradiated or irradiated CFSE‐stained cells for 3 h in vitro. The representative FCM images and method of gating CD11c+CFSE+ cells are shown. (B) BMDCs were cultured with non‐irradiated or irradiated cells for 24 h in vitro. The representative FCM images and method of gating CD80+ or CD86+ cells are shown. Figure S3: Assessment of ddC‐mediated mitochondrial depletion in cells and vaccine safety. (A) The ratio of mtDNA to nuclear DNA was quantified by real‐time PCR, with untreated Hepa1‐6 cells serving as the control. (B) On days 0, 14 and 28, mice were administered subcutaneously with PBS, 5 × 10^5^ irradiated Hepa 1‐6 cells, or 5 × 10^5^ irradiated Hepa 1‐6‐mGM‐CSF cells. Body weight was measured on day 1 post‐immunisation. (C, D) On day 7 following the third immunisation, serum and major organs (heart, liver, spleen, lung and kidney) were collected. (C) The biochemical parameters and (D) hematoxylin and eosin (H&E) staining of major organs were performed. (A) n = 3 per group. (B–D) n = 5 per group. Data are shown as mean ± SEM. ****p < 0.0001, ns, not statistically significant. [file CPR-9999-e70198-s001.docx]

**Supplementary Materials**

**Irradiated Liver Cancer Cell Vaccine Transfected With GM‐CSF Induces Specific and Long‐Lasting Anti‐tumour Immunity Through the Synergistic Effect of Oxidised mtDNA and GM‐CSF**

Zhiruo Song^1^, Yujie Jiang^1^, Yu Zhang^1^, Danyi Ao^1^, Chunjun Ye^1^, Xiya Huang^1^, Yingqiong Zhou^1^, Hanle Yang^1^, Ruolan Xia^1^, Jiayuan Ai^1^, Dandan Wan^1^, Aiping Tong^1^, Yuquan Wei^1^, Xuemei He^1, ⁎^, Aqu Alu^1, ⁎^, Xiawei Wei^1, ⁎^

^1^Laboratory of Aging Research and Cancer Drug Target, State Key Laboratory of Biotherapy and Cancer Center, National Clinical Research Center for Geriatrics, West China Hospital, Sichuan University, Chengdu 610041, China.

^⁎^Corresponding authors: Xuemei He ([hexuemei1116@126.com](mailto:hexuemei1116@126.com)), Aqu Alu ([aluaqu@qq.com](mailto:aluaqu@qq.com)) and Xiawei Wei (xiaweiwei@scu.edu.cn)

**Includes:**

**Supplementary Table 1 and 2**

**Supplementary Figure 1-3**

**Tables**

**Supplementary Table 1. Primers used in qPCR and RT-qPCR**

| Gene name | Forward sequences  Reverse sequences |
| --- | --- |
| β-actin | AGAGGGAAATCGTGCGTGAC  CAATAGTGATGACCTGGCCGT |
| mGM-CSF | GCTCTAGAAGATCACCGGCGAAGGA  TATGCGGCCGCTTCCTCATTTTTGGCC |
| mtDNA (cytochrome c oxidase I) | GCCCCAGATATAGCATTCCC  GTTCATCCTGTTCCTGCTCC |
| nDNA (18S ribosomal RNA) | TAGAGGGACAAGTGGCGTTC  CGCTGAGCCAGTCAGTGT |
| *mIrf7* | CAATTCAGGGGATCCAGTTG  AGCATTGCTGAGGCTCACTT |
| *mDdx58* | GAGTACCACTTAAAGCCAGAG  AATCCATTTCTTCAGAGCATCC |
| *mIfna4* | CTTTCCTCATGATCCTGGTAATGAT  AATCCAAAATCCTTCCTGTCCTTC |
| *mIfnb1* | CCCTATGGAGATGACGGAGA  CCCAGTGCTGGAGAAATTGT |
| *mIsg15* | CTAGAGCTAGAGCCTGCAG  AGTTAGTCACGGACACCAG |
| *mCxcl10* | CCAAGTGCTGCCGTCATTTTC  GGCTCGCAGGGATGATTTCAA |
| *mTnfa* | CATCTTCTCAAAATTCGAGTGACAA  CCAGCTGCTCCTCCACTTG |
| *mIl12* | CCATTGAACTGGCGTTGGAAG  ACTTGAGGGAGAAGTAGGAATGG |

**Supplementary Table 2. The information of antibodies.**

| **Antibody** | **Supplier** | **Cat. No.** | **Application** | **Dilution** |
| --- | --- | --- | --- | --- |
| PerCP-Cy5.5 anti-mouse CD45 antibody | Biolegend | 103132 | FCM | 1:100 |
| APC anti-mouse CD11c antibody | Biolegend | 117310 | FCM | 1:100 |
| PE-Cy7 anti-mouse MHC-Ⅱ antibody | Biolegend | 107630 | FCM | 1:100 |
| PE anti-mouse CD80 antibody | Biolegend | 104708 | FCM | 1:100 |
| FITC anti-mouse CD86 antibody | Biolegend | 105006 | FCM | 1:100 |
| PerCP-Cy5.5 anti-mouse CD3 antibody | Biolegend | 100218 | FCM | 1:100 |
| APC anti-mouse CD4 antibody | Biolegend | 100412 | FCM | 1:100 |
| BV510 anti-mouse CD8a antibody | Biolegend | 100752 | FCM | 1:100 |
| PE-Cy7 anti-mouse CD69 antibody | Biolegend | 104512 | FCM | 1:100 |
| FITC anti-mouse CD4 antibody | Biolegend | 100406 | FCM | 1:100 |
| APC anti-mouse CD8a antibody | Biolegend | 100712 | FCM | 1:100 |
| BV510 anti-mouse/human CD44 antibody | Biolegend | 103044 | FCM | 1:100 |
| PE-Cy7 anti-human/mouse Granzyme B recombinant antibody | Biolegend | 372214 | FCM | 1:100 |
| PE anti-mouse TNF-α antibody | Biolegend | 506306 | FCM | 1:100 |
| PE anti-mouse CD44 antibody | Biolegend | 103008 | FCM | 1:100 |
| BV421 anti-mouse CD62L antibody | Biolegend | 104436 | FCM | 1:100 |
| BV510 anti-mouse CD4 antibody | Biolegend | 100449 | FCM | 1:100 |
| BV421 anti-mouse CD279 (PD-1) antibody | Biolegend | 135218 | FCM | 1:100 |
| FITC anti-mouse CD185 (CXCR5) antibody | Biolegend | 145520 | FCM | 1:100 |
| PE anti-mouse CD19 antibody | Biolegend | 152408 | FCM | 1:100 |
| BV421 anti-mouse/human GL7 antigen antibody | Biolegend | 144614 | FCM | 1:100 |
| FITC anti-mouse CD95 (Fas) antibody | Biolegend | 152606 | FCM | 1:100 |
| APC anti-mouse CD25 antibody | Biolegend | 102012 | FCM | 1:100 |
| PE anti-mouse/rat/human Foxp3 antibody | Biolegend | 320008 | FCM | 1:100 |
| PE anti-mouse CD3 antibody | Biolegend | 100206 | FCM | 1:100 |
| BV421 anti-mouse CD4 antibody | Biolegend | 100563 | FCM | 1:100 |
| BV421 anti-mouse/human CD11b antibody | Biolegend | 101251 | FCM | 1:100 |
| PE anti-mouse F4/80 antibody | Biolegend | 123110 | FCM | 1:100 |
| PE anti-mouse CD45 antibody | Biolegend | 103106 | FCM | 1:100 |
| PE-Cy7 anti-mouse/human CD11b antibody | Biolegend | 101216 | FCM | 1:100 |
| PerCP-Cy5.5 anti-mouse Ly-6G/Ly-6C (Gr-1) antibody | Biolegend | 108428 | FCM | 1:100 |
| PE anti-mouse CD8a antibody | Biolegend | 100708 | FCM | 1:100 |
| BV510 anti-mouse CD11c antibody | Biolegend | 117338 | FCM | 1:100 |
| APC anti-mouse CD40 antibody | Biolegend | 124612 | FCM | 1:100 |
| PE anti-mouse CD45 antibody | Biolegend | 103106 | FCM | 1:100 |
| PE anti-mouse CD80 antibody | Biolegend | 104708 | FCM | 1:100 |
| BV421 anti-mouse CD40 antibody | Biolegend | 124641 | FCM | 1:100 |
| PerCP-Cy5.5 anti-mouse CD197 (CCR7) antibody | Biolegend | 120116 | FCM | 1:100 |
| PE-Cy7 anti-mouse CD11c antibody | Biolegend | 117318 | FCM | 1:100 |
| APC anti-mouse CD80 antibody | Biolegend | 104714 | FCM | 1:100 |
| FITC anti-mouse CD8a antibody | Biolegend | 100706 | FCM | 1:100 |
| Anti-caspase-3 antibody | Cell Signaling Technology | 9664 | FCM | 1:400 |
| Anti-8-OHdG antibody | Abcam | Ab62623 | FCM and IF | 1:500 |
| Anti-TOMM20 antibody | Abcam | Ab186734 | IF | 1:250 |
| Anti-phospho-TBK1/NAK (Ser172) rabbit antibody | Cell Signaling Technology | 5483 | WB | 1:1000 |
| Anti-TBK1/NAK rabbit antibody | Cell Signaling Technology | 3013 | WB | 1:1000 |
| Anti-phospho-IRF-3 (Ser396) rabbit antibody | Cell Signaling Technology | 4947 | WB | 1:1000 |
| Anti-IRF3 antibody rabbit antibody | Cell Signaling Technology | 4302 | WB | 1:1000 |
| Anti-phospho-Jak2 (Tyr1007/1008) rabbit antibody | Cell Signaling Technology | 3771 | WB | 1:1000 |
| Anti-Jak2 rabbit antibody | Cell Signaling Technology | 3230 | WB | 1:1000 |
| Anti-phospho-Stat3 (Tyr705) rabbit antibody | Cell Signaling Technology | 9145 | WB | 1:1000 |
| Anti-Stat3 mouse antibody | Cell Signaling Technology | 9139 | WB | 1:1000 |
| Anti-GAPDH rabbit antibody | HUABIO | ET1601-4 | WB | 1:50000 |

Abbreviations: FCM, flow cytometry; IF, immunofluorescence; WB, western blot

**Figures**

**Supplementary Figure 1-3**

**
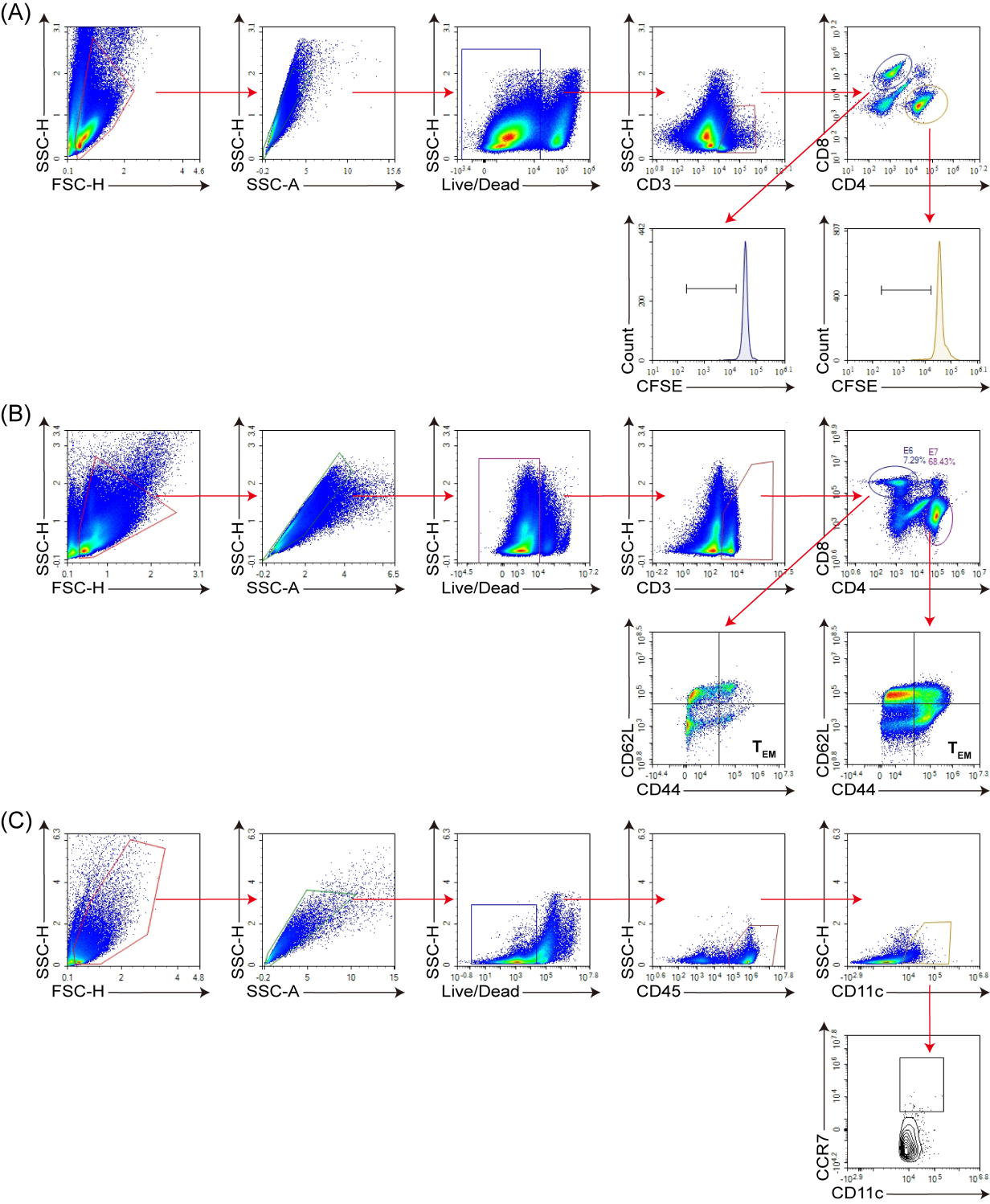
**

**Supplementary Figure 1 The gating method for T cells in spleen and DCs in lymph node.** (A) The representative FCM images and method of gating proliferative CD4^+^ and CD8^+^ T cells (CFSE^low^) in splenic lymphocytes of immunised mice after being stimulated by irradiated Hepa 1-6 cells for 72 h. (B) The representative FCM images and method of gating T_EM_ in spleens of immunised mice challenged with 5 × 10^6^ Hepa 1-6 cells. (C) The representative FCM images and method of gating CD11c^+^CCR7^+^ cells in popliteal LNs are shown. Abbreviations: FCM, flow cytometry; LN, lymph node.

**
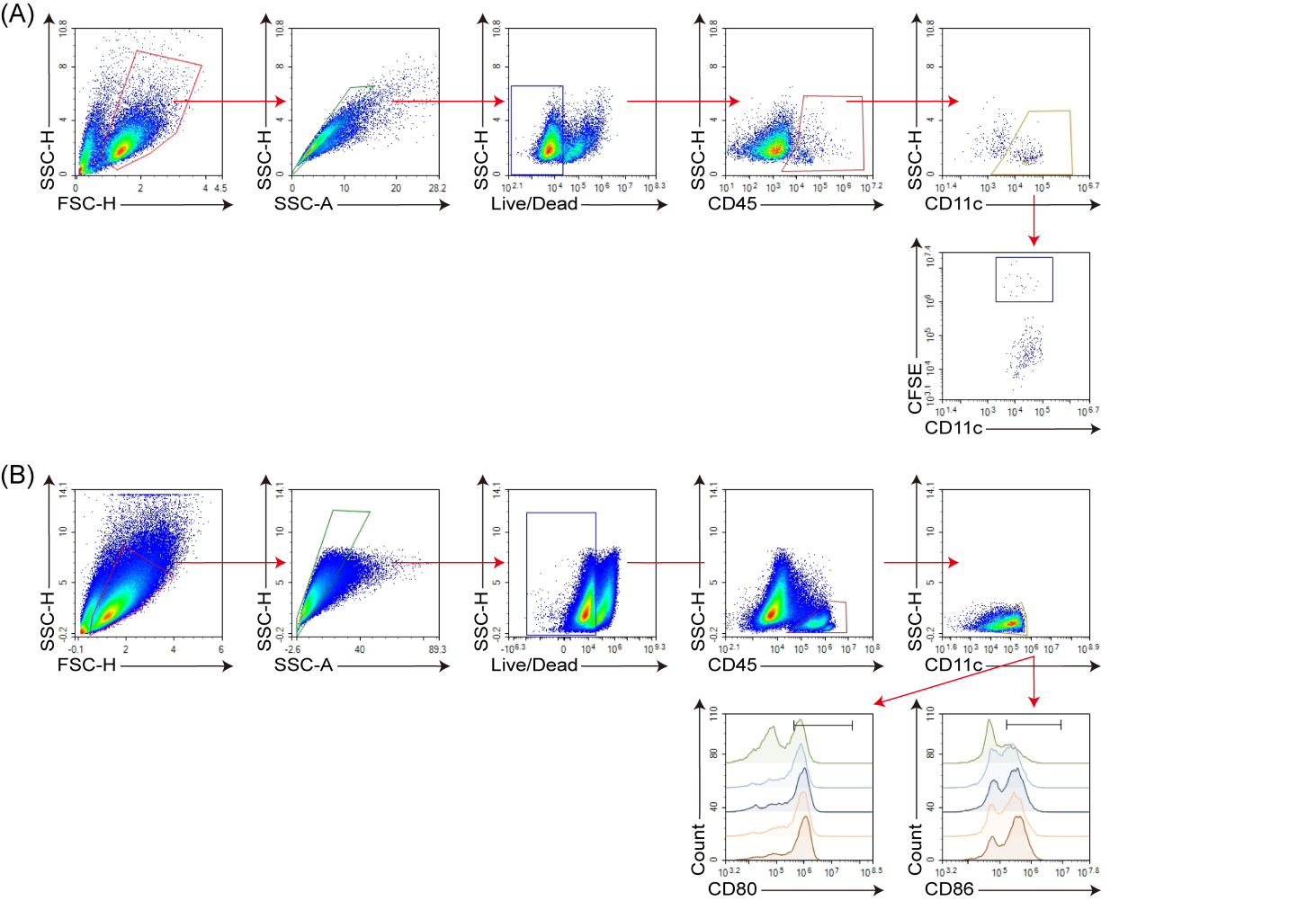
**

**Supplementary Figure 2 The gating method for BMDCs.** (A) BMDCs were cultured with nonirradiated or irradiated CFSE-stained cells for 3 h in vitro. The representative FCM images and method of gating CD11c^+^CFSE^+^ cells are shown. (B) BMDCs were cultured with nonirradiated or irradiated cells for 24 h in vitro. The representative FCM images and method of gating CD80^+^ or CD86^+^ cells are shown.

**
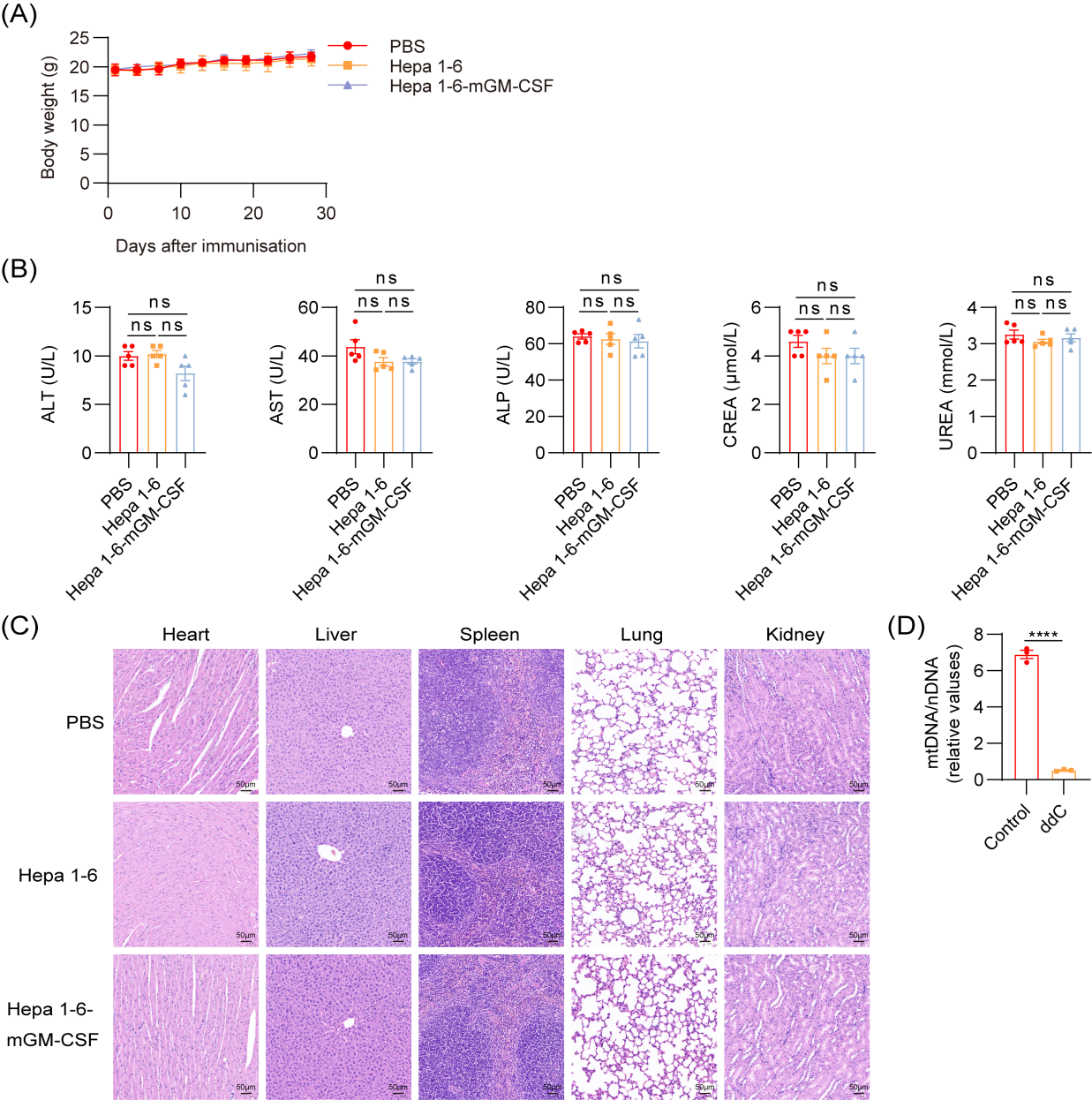
**

**Supplementary Figure 3** **Assessment of ddC-mediated mitochondrial depletion in cells and vaccine safety.** (A) The ratio of mtDNA to nuclear DNA was quantified by real-time PCR, with untreated Hepa 1-6 cells serving as the control. (B) On days 0, 14, and 28, mice were administered subcutaneously with PBS, 5 × 10^5^ irradiated Hepa 1-6 cells, or 5 × 10^5^ irradiated Hepa 1-6-mGM-CSF cells. Body weight was measured on day 1 post-immunisation. (C, D) On day 7 following the third immunisation, serum and major organs (heart, liver, spleen, lung, and kidney) were collected. (C) The biochemical parameters and (D) hematoxylin and eosin (H&E) staining of major organs were performed. (A) n = 3 per group. (B-D) n = 5 per group. Data are shown as mean ± SEM. **** p < 0.0001, ns: not statistically significant.
